# Prevalence and Prognostic Significance of Bradyarrhythmias in Patients Screened for Atrial Fibrillation vs Usual Care: Post Hoc Analysis of the LOOP Randomized Clinical Trial

**DOI:** 10.1001/jamacardio.2022.5526

**Published:** 2023-02-15

**Authors:** Søren Zöga Diederichsen, Lucas Yixi Xing, Diana My Frodi, Emilie Katrine Kongebro, Ketil Jørgen Haugan, Claus Graff, Søren Højberg, Derk Krieger, Axel Brandes, Lars Køber, Jesper Hastrup Svendsen

**Affiliations:** 1 Department of Cardiology, Copenhagen University Hospital–Rigshospitalet, Copenhagen, Denmark; 2 Department of Cardiology, Zealand University Hospital Roskilde, Roskilde, Denmark; 3 Department of Health Science and Technology, Aalborg University, Aalborg, Denmark; 4 Department of Cardiology, Copenhagen University Hospital–Bispebjerg, Copenhagen, Denmark; 5 Stroke Unit, Mediclinic City Hospital, Dubai, United Arab Emirates; 6 Department of Cardiology, Odense University Hospital, Odense, Denmark; 7 Department of Clinical Research, Faculty of Health Sciences, University of Southern Denmark, Odense, Denmark; 8 Department of Internal Medicine–Cardiology, University Hospital of Southern Denmark–Esbjerg, Esbjerg, Denmark; 9 Department of Clinical Medicine, Faculty of Health and Medical Sciences, University of Copenhagen, Copenhagen, Denmark

## Abstract

**Importance:**

There is increasing interest in heart rhythm monitoring and technologies to detect subclinical atrial fibrillation (AF), which may lead to incidental diagnosis of bradyarrhythmias.

**Objective:**

To assess bradyarrhythmia prevalence and prognostic significance in persons screened for AF using implantable loop recorder (ILR) compared with unscreened persons.

**Design, Setting, and Participants:**

This was a post hoc analysis of the Implantable Loop Recorder Detection of Atrial Fibrillation to Prevent Stroke (LOOP) randomized clinical trial, which took place in 4 sites in Denmark. Participants were 70 years or older without known AF but diagnosed with at least 1 of the following: hypertension, diabetes, heart failure, or prior stroke. Participants were recruited by letter invitation between January 31, 2014, and May 17, 2016. The median (IQR) follow-up period was 65 (59-70) months. Analysis took place between February and June 2022.

**Interventions:**

ILR screening for AF with treatment of any bradyarrhythmia left to the discretion of the treating physician (ILR group) vs usual care (control group).

**Main Outcomes and Measures:**

Adjudicated bradyarrhythmia episodes, pacemaker implantation, syncope, and sudden cardiovascular death.

**Results:**

A total of 6004 participants were randomized (mean [SD] age, 75 [4.1] years; 2837 [47.3%] female; 5444 [90.7%] with hypertension; 1224 [20.4%] with prior syncope), 4503 to control and 1501 to ILR. Bradyarrhythmia was diagnosed in 172 participants (3.8%) in the control group vs 312 participants (20.8%) in the ILR group (hazard ratio [HR], 6.21 [95% CI, 5.15-7.48]; *P* < .001), and these were asymptomatic in 41 participants (23.8%) vs 249 participants (79.8%), respectively. The most common bradyarrhythmia was sinus node dysfunction followed by high-grade atrioventricular block. Risk factors for bradyarrhythmia included higher age, male sex, and prior syncope. A pacemaker was implanted in 132 participants (2.9%) vs 67 (4.5%) (HR, 1.53 [95% CI, 1.14-2.06]; *P* < .001), syncope occurred in 120 (2.7%) vs 33 (2.2%) (HR, 0.83 [95% CI, 0.56-1.22]; *P* = .34), and sudden cardiovascular death occurred in 49 (1.1%) vs 18 (1.2%) (HR, 1.11 [95% CI, 0.64-1.90]; *P* = .71) in the control and ILR groups, respectively. Bradyarrhythmias were associated with subsequent syncope, cardiovascular death, and all-cause death, with no interaction between bradyarrhythmia and randomization group.

**Conclusions and Relevance:**

More than 1 in 5 persons older than 70 years with cardiovascular risk factors can be diagnosed with bradyarrhythmias when long-term continous monitoring for AF is applied. In this study, ILR screening led to a 6-fold increase in bradyarrhythmia diagnoses and a significant increase in pacemaker implantations compared with usual care but no change in the risk of syncope or sudden death.

## Key Points

**Question:**

In a future of increased heart rhythm monitoring, what is the expected prevalence and prognostic significance of incidentally diagnosed bradyarrhythmias?

**Findings:**

In this post hoc analysis of a randomized clinical trial of persons older than 70 years undergoing implantable loop recorder screening for unknown atrial fibrillation vs usual care, screening was associated with incidental diagnosis of sinus node dysfunction or atrioventricular block in 1 in 5 persons and increased pacemaker implantations, but there was no change in the risk of syncope or sudden death.

**Meaning:**

Bradyarrhythmias are highly common in older persons, and while these arrhythmias may constitute risk markers, their detection and treatment is not associated with reduced incidence of clinical outcomes.

## Teaser

This ad hoc analysis of a randomized clinical trial assesses bradyarrhythmia prevalence and prognostic significance in persons screened for atrial fibrillation using implantable loop recorder compared with unscreened persons.

## Introduction

Recent years have seen an increased interest in heart rhythm monitoring and a surge of new technologies to detect arrhythmias.^1,2,3^ Much interest has focused on tools to detect subclinical atrial fibrillation (AF)^4,5,6,7^ or help manage or diagnose patients with palpitations,^8,9,10^ whereas data on sinus node dysfunction (SND) and atrioventricular block (AVB) are scarce. Similarly to AF, the detection of bradyarrhythmias should increase with monitoring duration and may constitute an incidental, subclinical finding.^11^ Given increased heart rhythm monitoring and consumer-led screening, evidence on the underlying prevalence and prognostic significance of bradyarrhythmias could help guide clinical decision-making.

Using a randomized trial of persons 70 years or older recruited outside the hospital setting to receive implantable loop recorder (ILR) monitoring vs usual care, we aimed to assess the incidental diagnosis of bradyarrhythmia and its prognostic implications in screened persons compared with unscreened persons.

## Methods

### Study Design

The current study is a post hoc analysis of the Implantable Loop Recorder Detection of Atrial Fibrillation to Prevent Stroke (LOOP) study, a randomized clinical trial investigating stroke prevention by means of ILR screening for AF in persons with risk factors. The main finding was that the screening resulted in a 3-fold increase in diagnosis of AF and initiation of anticoagulation but did not result in a significant reduction in stroke.^12^ The methodology has been reported previously,^13^ and the trial protocol is available in Supplement 1. Briefly, a random sample of persons 70 years or older and diagnosed with at least 1 of 4 conditions (hypertension, diabetes, heart failure, or previous stroke) but with no history of AF or cardiac implantable electronic device was identified through administrative registries and invited by letter to participate. Data on race and ethnicity were not collected. Eligible participants were randomized in a 1:3 ratio to receive an ILR or usual care (control group) from January 31, 2014, to May 17, 2016. In the ILR group, a Reveal LINQ (Medtronic) was implanted, preferably within 1 month, and remote monitoring with daily review of any arrhythmias continued until death, device removal, or end of service (minimum, 3 years). A threshold of 30 beats per minute or less during 4 beats or more or asystole lasting 3 seconds or longer was used to detect bradyarrhythmias in the ILR group, while, importantly, treatment in case of bradyarrhythmia diagnosis was left to the discretion of the treating physician in both groups. Written informed consent was obtained from all participants prior to enrollment. The trial was approved by the Capital Region of Denmark Research Ethics Committee (H-4-2013-025) and Data Protection Agency (2007-58-0015) and registered at ClinicalTrials.gov (NCT02036450).

In the ILR group, apart from the remote monitoring, outcomes were collected during annual on-site study visits for the first 3 years followed by annual telephone contact with lookup in records from all hospital admissions, outpatient visits, and drug prescriptions. The control group underwent a single on-site study visit at year 3 along with annual telephone contact. At the end of the main trial, all participants still alive underwent a final assessment by lookup in all health records.

### Outcomes

The outcomes of interest for the current analysis were bradyarrhythmia, AF, syncope, pacemaker implantation, sudden cardiovascular death, cardiovascular death, and all-cause death. Bradyarrhythmias were adjudicated by an experienced physician with access to electrocardiography and health records. In cases of suspected bradyarrhythmia during remote monitoring, a clinician phoned the participant to collect further details including symptoms. Physiological findings were discarded based the physician’s best judgment. This included asymptomatic episodes of sinus bradycardia, pause, arrest, or first-degree AVB or second-degree AVB Mobitz type I during sleep with asystole not lasting longer than 3.5 seconds and heart rate not dropping below 30 beats per minute during 8 consecutive beats. Adjudicated bradyarrhythmias were classified as one of (1) SND defined as inadequate heart rate compared with physiologic need due to sinus bradycardia, pause or arrest, exit block, or chronotropic incompetence; (2) low-grade AVB defined as first- or second-degree heart block with a P:QRS ratio of 2:1 or lower; or (3) high-grade AVB defined as complete heart block or second-degree heart block with a P:QRS ratio of at least 3:1.^14^ The first bradyarrhythmia episode per participant was adjudicated and its associated symptoms and treatment recorded, whereas any recurrent bradyarrhythmia episodes were also adjudicated if classified or treated differently than the incident episode. AF diagnoses were adjudicated as part of the main trial.^13^ Syncope was adjudicated by an experienced physician and was defined as a sudden and transient complete loss of consciousness with inability to maintain upright posture, without seizure or preceding trauma.^15^ All pacemaker implantations were recorded, while implanted cardioverter-defibrillator or cardiac resynchronization therapy without bradyarrhythmia as an indication were not considered. Deaths were adjudicated according to cardiovascular death and sudden death.

### Statistics

For summary statistics, continuous variables were presented as mean (SD) and median (IQR) for normally and nonnormally distributed variables and groupwise compared using *t* tests and Wilcoxon rank-sum tests, respectively, while categorical variables were presented as frequency (percentage) and groupwise compared by χ^2^ tests. Event rates were presented as events per 100 person-years (95% CI) and hazard ratios (HR) as HR (95% CI).

Time-to-event analyses were performed separately for the outcomes of bradyarrhythmia, pacemaker implantation, syncope, and sudden cardiovascular death comparing the control group and the ILR group. The highest-grade bradyarrhythmia was used for participants diagnosed with more than 1 subtype. Time to event was defined as time from baseline to first event or censoring, with right censoring at the end of follow-up or death. Cumulative incidences were calculated and compared using the Aalen-Johansen estimator to account for the competing risk of death.

Within each randomization group, the diagnosis of AF was analyzed as a risk factor for bradyarrhythmia and vice versa, the diagnosis of bradyarrhythmia was analyzed as a risk factor for syncope, sudden cardiovascular death, cardiovascular death, or all-cause death, and baseline variables were analyzed as risk factors for diagnosis of bradyarrhythmia. The analyses of AF as a risk factor for bradyarrhythmia comprised time-dependent models defining time to event by 2 periods in participants with the exposure (AF): time from baseline to start of the exposure (diagnosis of AF) and time from start of the exposure to outcome (diagnosis of bradyarrhythmia, death, or censoring). The association was then assessed using univariate cause-specific Cox proportional hazards models with multivariate adjustment retaining age, sex, and comorbidities used as inclusion criteria. An unadjusted model including both groups was tested to assess interaction between AF and randomization group (usual care–detected vs ILR-detected arrhythmia). The same methodology was applied to analyze bradyarrhythmia as a risk factor for AF, syncope, sudden cardiovascular death, cardiovascular death, or all-cause death. The analyses of baseline variables as risk factors for the diagnosis of bradyarrhythmia comprised cause-specific Cox proportional hazards models using time from baseline to diagnosis of bradyarrhythmia, death, or censoring with multivariate adjustment retaining age, sex, and comorbidities used as inclusion criteria along with any baseline variables univariately associated with time to diagnosis of bradyarrhythmia in either group. An unadjusted model including both groups was tested to assess interaction between randomization group and each risk factor identified.

Sensitivity analyses were performed excluding ILR participants who did not receive the ILR using time of implantation as baseline. The proportional-hazards assumption was assessed with Schoenfeld residuals. A 2-sided *P* value less than .05 was considered statistically significant. Analysis took place between February and June 2022.

## Results

### Study Overview

Overall, 6004 participants were randomized: 4503 to the control group and 1501 to the ILR group. The mean (SD) age was 75 (4.1) years and 2837 (47%) were female. The Table presents baseline characteristics according to randomization group. In the ILR group, 1420 participants (94.6%) received the ILR, and the median (IQR) monitoring duration was 39.0 (36.1-41.4) months.

**Table.** Baseline Characteristics

Characteristic	No. (%)	
	Control (n = 4503)	ILR (n = 1501)
Female	2128 (47.3)	709 (47.2)
Male	2375 (52.7)	792 (52.8)
Age, mean (SD), y	74.7 (4.1)	74.7 (4.1)
Hypertension	4066 (90.3)	1378 (91.8)
Diabetes	1288 (28.6)	422 (28.1)
Heart failure	199 (4.4)	67 (4.5)
Prior stroke, TIA, or SAE	1139 (25.3)	370 (24.7)
Prior AMI, CABG, or PCI	614 (13.6)	177 (11.8)
Prior CABG	250 (5.6)	87 (5.8)
Valvular heart disease	181 (4.0)	63 (4.2)
Prior syncope	924 (20.5)	300 (20.0)
CHA_2_DS_2_-VASc score, median (IQR)	4 (3-4)	4 (3-4)
2	588 (13.1)	202 (13.5)
3	1494 (33.2)	513 (34.2)
4	1325 (29.4)	419 (27.9)
5	687 (15.3)	244 (16.3)
≥6	409 (9.8)	123 (8.2)
Medical treatment		
β-Blockers	1172 (26.0)	354 (23.6)
Nondihydropyridine calcium blockers	97 (2.2)	44 (2.9)
Calcium blockers	1684 (37.4)	562 (37.4)
RA inhibitors	2999 (66.6)	991 (66.0)
Statins	2621 (58.2)	879 (58.6)
Diuretics	1511 (33.6)	495 (33.0)
Platelet inhibitors	2204 (48.9)	702 (46.8)
Insulins	354 (7.9)	124 (8.3)
Other antidiabetics	959 (21.3)	328 (21.9)
Physical evaluation, mean (SD)		
BMI	27.6 (4.5)	27.8 (4.7)
Systolic BP, mm Hg	149.8 (19.5)	150.6 (19.2)
Diastolic BP, mm Hg	83.9 (11.3)	84.7 (11.1)
Resting sinus rate, beats/min	71.3 (12.5)	71.6 (12.1)

Abbreviations: AMI, acute myocardial infarction; BMI, body mass index (calculated as weight in kilograms divided by height in meters squared); BP, blood pressure; CABG, coronary artery bypass graft; ILR, implantable loop recorder; PCI, percutaneous coronary intervention; RA, renin-angiotensin; SAE, systemic arterial embolism; TIA, transient ischemic attack.

Follow-up data were available through January 2021 with a median (IQR) follow-up period of 64.5 (59.3-69.8) months, and no participants were lost to follow-up. AF was diagnosed in 1057 participants (17.1%) (550 [12.2%] in the control group and 477 [31.8%] in the ILR group).^12^ A total of 675 deaths occurred with an overall incidence rate of 2.16 (95% CI, 2.00-2.33) per 100 person-years, and 200 cardiovascular deaths occurred with an overall incidence rate of 0.64 (95% CI, 0.55-0.73). A total of 67 sudden cardiovascular deaths occurred (control group: 49 [1.1%]; incidence rate, 0.21 [95% CI, 0.15-0.28] vs ILR group: 18 [1.2%]; incidence rate, 0.23 [95% CI, 0.14-0.37]; HR, 1.11 [95% CI, 0.64-1.90]; *P* = .71; eFigure in Supplement 2).

### Bradyarrhythmia Types, Symptoms, and Treatment

**Figure 1.**
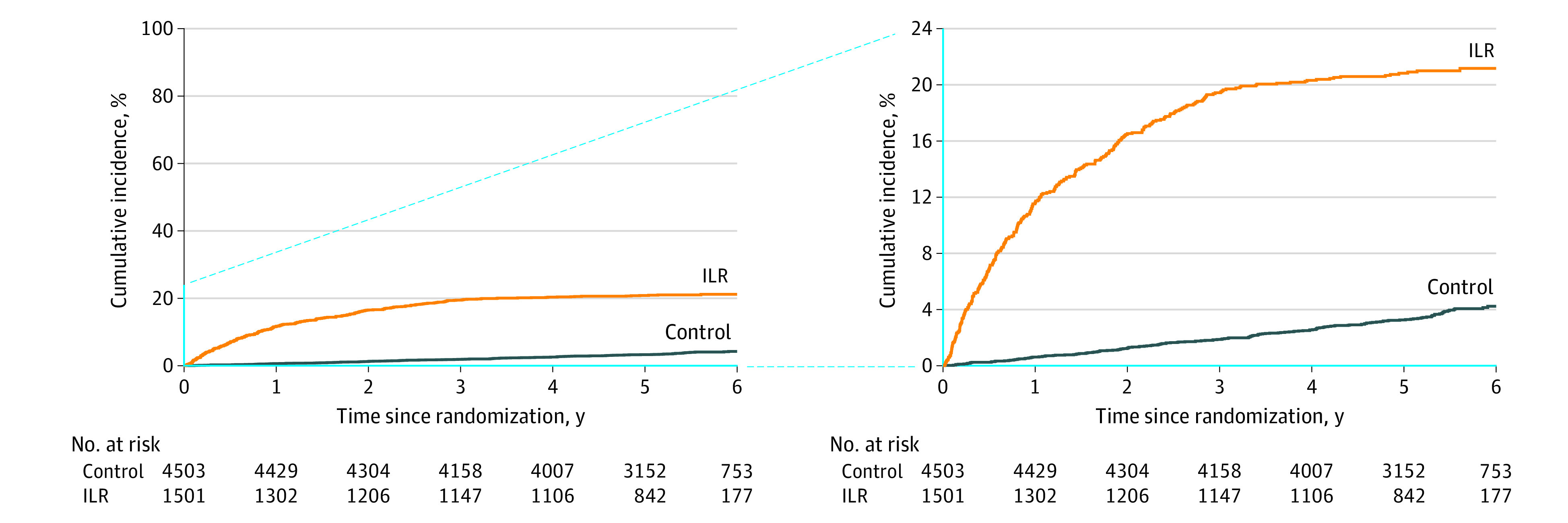
Bradyarrhythmia Detection Time to first event curves (with inset) for diagnosis of bradyarrhythmia by randomization group are displayed. ILR indicates implantable loop recorder.

A total of 484 participants (8.1%) were diagnosed with bradyarrhythmia (control group: 172 [3.8%]; incidence rate, 0.75 [95% CI, 0.64-0.87] vs ILR group: 312 [20.8%]; incidence rate, 4.76 [95% CI, 4.20-5.32]; HR, 6.21 [95% CI, 5.15-7.48]; *P* < .001, Figure 1), while the incidence rate was 13.12 (95% CI, 11.24-15.23) during the first year after randomization in those who received an ILR. None of the participants who refused ILR were diagnosed with bradyarrhythmia.

**Figure 2.**
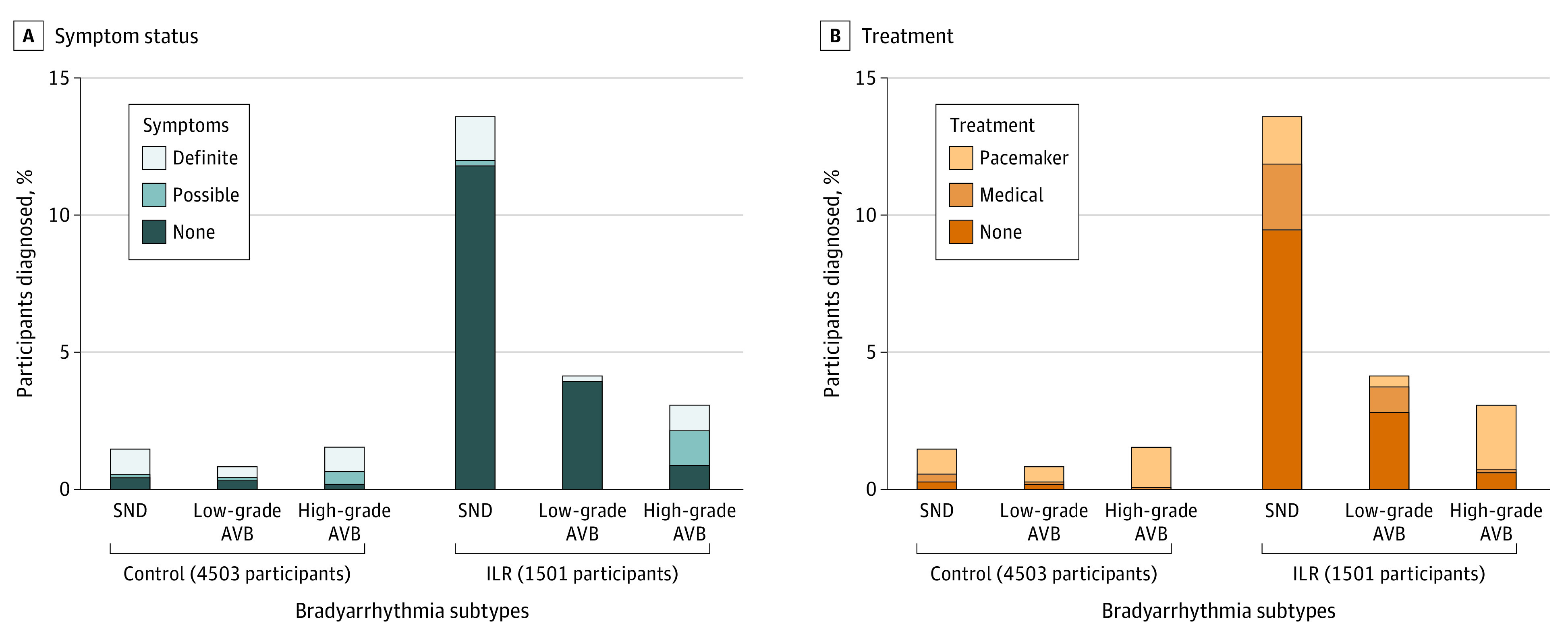
Bradyarrhythmia Subtypes, Symptoms, and Treatment Bradyarrhythmia diagnoses with symptom status (A) and treatment (B) by randomization group are displayed, counting the highest-grade event in participants diagnosed with more than 1 subtype (21 participants [0.5%] in the control group and 11 [0.7%] in the ILR group). Medical treatment includes drug dose adjustments or discontinuation or treatment of concomitant conditions. AVB indicates atrioventricular block; ILR, implantable loop recorder; SND, sinus node dysfunction.

SND was diagnosed in 270 participants (4.5%) who did not have higher grade bradyarrhythmia (control group: 66 [1.5%] vs ILR group: 204 [13.6%]), whereas low-grade AVB was diagnosed in 99 participants (1.6%) (control group: 37 [0.8%] vs ILR group: 62 [4.1%]) and high-grade AVB was diagnosed in 115 participants (1.9%) (control group: 69 [1.5%] vs ILR group: 46 [3.1%]; Figure 2). A total of 141 participants (2.4%) had definitely symptomatic bradyarrhythmias (control group: 100 [2.2%] vs ILR group: 41 [2.7%]), whereas 53 participants (0.8%) had bradyarrhythmias with possible symptoms (control group: 31 [0.7%] vs ILR group: 22 [1.5%]). Completely asymptomatic bradyarrhythmia was seen in 290 participants (4.8%) (control group: 41 [0.9%] vs ILR group: 249 [16.6%]). Symptoms were absent in 196 participants with SND (72.6% of all with SND; control group: 19 [28.8%] vs ILR group: 177 [86.8%]), 73 participants with low-grade AVB (73.7% of all with low-grade AVB; control group: 14 [37.8%] vs ILR group: 59 [95.1%]), and 21 participants (18.3% of all with high-grade AVB; control group: 8 [11.5%] vs ILR group: 13 [28.3%]).

A total of 67 participants with SND received a pacemaker (24.8% of all with SND; control group: 41 [62.1%] vs ILR group: 26 [12.7%]). A total of 31 participants with low-grade AVB received a pacemaker (31.3% of all with low-grade AVB; control group: 25 [67.6%] vs ILR group: 6 [9.7%]). A total of 101 participants with high-grade AVB received a pacemaker (87.8% of all with high-grade AVB; control group: 56 [95.7%] vs ILR group: 35 [76.1%]). Of 3 participants with usual care–detected high-grade AVB not treated with pacemaker, 2 occurred during noncardiac terminal illness. Of 11 participants with ILR-detected high-grade AVB not treated with pacemaker, 6 occurred during nighttime only and 2 during noncardiac terminal illness.

### Syncope and Pacemaker Implantation

**Figure 3.**
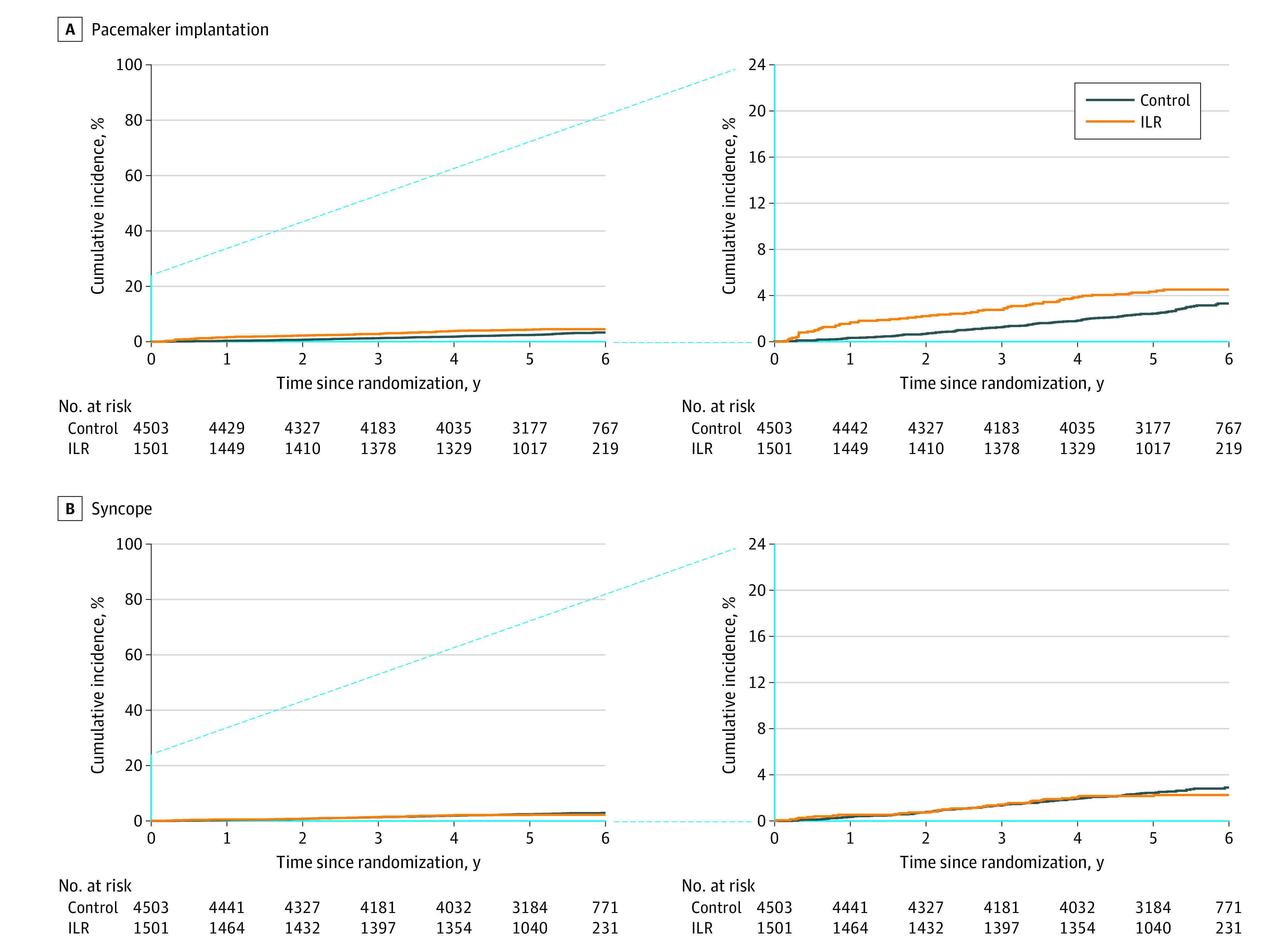
Pacemaker Implantation and Syncope Time to first event curves (with insets) for pacemaker implantation (A) and syncope (B) by randomization group. ILR indicates implantable loop recorder.

**Figure 4.**
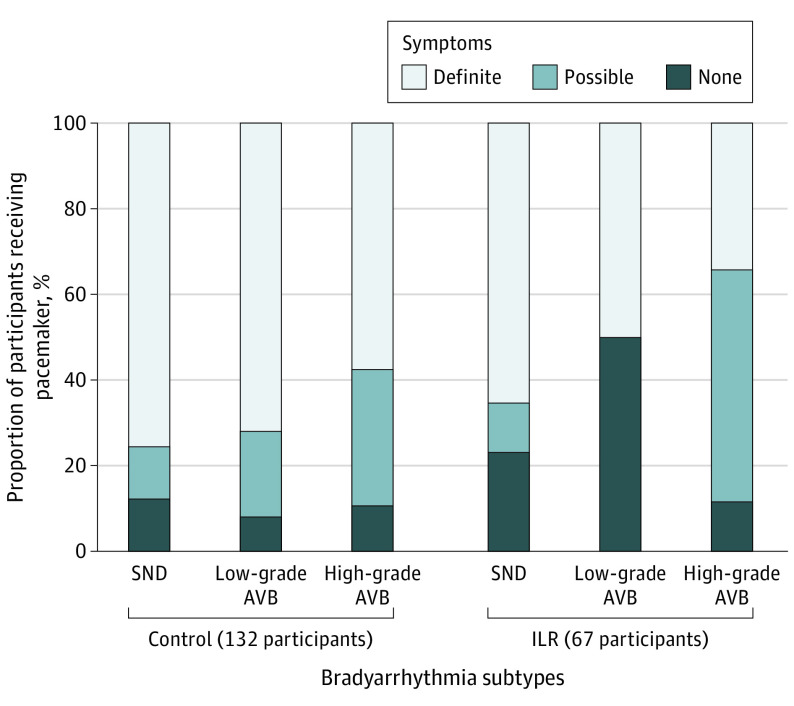
Symptoms in Participants Receiving Pacemaker Symptoms according to indication in participants receiving pacemaker by randomization group are displayed. AVB indicates atrioventricular block; ILR, implantable loop recorder; SND, sinus node dysfunction.

A total of 199 participants (3.3%) received a pacemaker during follow-up (control group: 132 [2.9%]; incidence rate, 0.57 [95% CI, 0.48-0.67] vs ILR group: 67 [4.5%]; incidence rate, 0.87 [95% CI, 0.67-1.11]; HR, 1.53 [95% CI, 1.14-2.06]; *P* < .001; Figure 3), while the incidence rate was 1.71 (95% CI, 1.10-2.55) during the first year after randomization in those who received an ILR. A total of 119 patients receiving a pacemaker were definitely symptomatic (59.8% of all receiving a pacemaker; control group: 87 [65.9%] vs ILR group: 32 [47.8%]), and 50 patients receiving a pacemaker for high-grade AVB were definitely symptomatic (49.5% of all receiving a pacemaker for high-grade AVB; control group: 48 [57.6%] vs ILR group: 12 [34.3%]; Figure 4).

A total of 153 participants (2.6%) experienced syncope (control group: 120 [2.7%]; incidence rate, 0.52 [95% CI, 0.43-0.62] vs ILR group: 33 [2.2%]; incidence rate, 0.43 [95% CI, 0.30-0.60]; HR, 0.83 [95% CI, 0.56-1.22]; *P* = .34). Of these, 68 (44.4%) subsequently received a pacemaker: 49 (40.8%) in the control group and 19 (57.6%) in the ILR group. One participant who refused ILR experienced syncope but was not diagnosed with arrhythmia nor received a pacemaker. Recurrent syncope was seen in 7 participants, all from the control group, 3 of whom were diagnosed with bradyarrhythmia at their second syncope and then received a pacemaker.

### Bradyarrhythmia and AF

The prevalence of bradyarrhythmia was higher in participants with AF (196 of 1057 [19.1%]) than without (288 of 4977 [5.8%]), and this was true for both randomization groups. The relative increase was larger in the control group in which 68 of 550 participants (12.4%) diagnosed with AF during follow-up were also diagnosed with bradyarrhythmia, compared with 104 of 3953 participants (2.6%) not diagnosed with AF (eTable 1 in Supplement 2). Among participants with concomitant AF and bradyarrhythmia, AF was diagnosed before bradyarrhythmia in 38 of 68 participants (55.6%) in the control group and 74 of 128 participants (58.6%) in the ILR group. The time-dependent analyses found that diagnosis of AF was associated with subsequent bradyarrhythmia in the control group only (control group: 5.69 [95% CI, 3.91-8.28]; *P* < .001 vs ILR group: 1.01 [95% CI, 0.78-1.31]; *P* = .93), whereas diagnosis of bradyarrhythmia was associated with subsequent AF in the control group but inversely in the ILR group (control group: 3.65 [95% CI, 3.35-5.66]; *P* < .001 vs ILR group: 0.72 [95% CI, 0.54-0.96]; *P* = .03). These associations remained significant after multivariate adjustment, and the analyses including the full study population found significant interactions between AF and randomization group as well as the bradyarrhythmia and randomization group, respectively (*P* for interaction <.001 in both settings). The sensitivity analyses did not change the findings.

### Bradyarrhythmia and Subsequent Outcomes

The number of participants diagnosed with bradyarrhythmia subsequently experiencing syncope was 18 (3.7% of all with bradyarrhythmia; control group: 7 [4.1%] vs ILR group: 11 [3.5%]), whereas 6 experienced sudden cardiovascular death (1.2% of all with bradyarrhythmia; control group: 1 [0.6%] vs ILR group: 5 [1.6%]), 23 cardiovascular death (4.8% of all with bradyarrhythmia control group: 10 [5.81%] vs ILR group: 13 [4.2%]), and 72 all-cause death (14.9% of all with bradyarrhythmia; control group: 25 [14.5%] vs ILR group: 47 [15.1%]). In both randomization groups, the time-dependent analyses found that diagnosis of bradyarrhythmia was associated with subsequent syncope (control group: HR, 5.20 [95% CI, 2.41-11.22]; *P* < .001 vs ILR group: HR, 2.57 [95% CI, 1.25-5.32]; *P* = .01), cardiovascular death (control group: HR, 4.81 [95% CI, 2.58-8.94]; *P* < .001 vs ILR group: HR, 3.13 [95% CI, 1.66-5.92]; *P* < .001), and all-cause death (control group: HR, 3.13 [95% CI, 1.66-5.92]; *P* < .001 vs ILR group: HR, 2.48 [95% CI, 1.77-3.46]; *P* < .001), and these associations remained significant after multivariate adjustment, while the analyses including the full study population found no interactions between bradyarrhythmia and randomization group (*P* for interaction = 0.28, 0.31, and 0.11, respectively). The was no association between bradyarrhythmia and sudden cardiovascular death in either group (control group: HR, 1.12 [95% CI, 0.15-8.14]; *P* = .91 vs ILR group: HR, 2.06 [95% CI, 0.73-5.80]; *P* = .17). The sensitivity analyses did not change the findings.

### Bradyarrhythmia Risk Factors

Older age, male sex, and prior syncope were associated with incident bradyarrhythmia in both randomization groups (eTable 2 in Supplement 2), whereas heart failure, prior coronary bypass graft, valvular heart disease, and lower resting sinus rate were also associated with incident bradyarrhythmia in the control group, and higher body mass index in the ILR group. Of medications, only platelet inhibitors were associated with bradyarrhythmia, and only in the ILR group, but not after adjustment for age and sex. The analysis including the full study population demonstrated a significant interaction between randomization group and age, sex, heart failure, valvular heart disease, and resting sinus rate, respectively, whereas there was no interaction between other variables. The sensitivity analyses did not change the findings.

## Discussion

To our knowledge, this is the first study to assess the incidental diagnosis of bradyarrhythmia, using data from a randomized clinical trial of persons older than 70 years recruited outside the hospital setting to undergo AF screening vs usual care. The key finding of this post hoc study was that the screening led to a 6-fold increase in bradyarrhythmia detection and a significant increase in pacemaker implantations compared with usual care, with no signal toward a change in the risk of syncope or sudden death. A substantial proportion of bradyarrhythmias were completely asymptomatic, which was even true for more advanced episodes. Bradyarrhythmias were independently associated with clinical outcomes but not more so for bradyarrhythmias detected by screening than usual care. Finally, bradyarrhythmias often coexisted with AF.

### Subclinical Bradyarrhythmia and Future Perspectives

Recent years have seen an increased interest in screening for AF.^11,13,16,17,18,19^ Given a future of increased heart rhythm monitoring inside and outside the clinical setting,^20^ bradyarrhythmias are likely to be detected more often, sometimes as an incidental finding. Knowledge about the underlying prevalence and prognostic significance could help guide decisions.

The digital age holds promise of early detection of a range of conditions. When detected at an asymptomatic, subclinical stage, questions arise to whether abnormal findings represent a clinical problem needing diagnosis and treatment or merely a risk marker without implications. Some conditions may be considered part of normal physiology or aging. These questions apply not only to heart rhythm monitoring but to a growing field of measurements instigated by clinicians, the industry, and consumers alike.

In the current trial, AF screening led to an excessive increase in diagnosis of SND and AVB compared with usual care and resulted in more pacemaker implantations but no improvement in clinical outcomes. The majority of added diagnoses from screening were left untreated without adverse consequence. Bradyarrhythmias were statistically associated with syncope and mortality, but, according to the interaction analysis, not more so for episodes detected by ILR than by usual care and not with mortality types that could possibly have been prevented by cardiac pacing (sudden cardiac death). The latter is relevant considering the often conservative treatment and supports the argument that bradyarrhythmias can be risk markers and not a disease themselves. Also, the much higher detection rate in the first year of ILR monitoring compared with subsequent years could indicate an underlying prevalence of SND or AVB even before randomization in the trial. Indeed, the increased detection in the ILR group compared with control was due to asymptomatic cases, and clinical outcomes were not impacted by these diagnoses and their management. Importantly, this trial cannot tell whether pacemakers had an association in the individual patient with bradyarrhythmia, but the screening and downstream interventions did not decrease overall rates of syncope or sudden cardiovascular death, and, as previously reported, not cardiovascular death or all-cause death either.^12^

### Association With Syncope and Pacemaker Implantations

Syncope was relatively rare but was more frequently followed by pacemaker implantation in the ILR group compared with control. The even lower occurrence of recurrent syncope could indicate that appropriate precautions were taken following the first syncope, whereas the lack of signal toward a difference in syncope across the randomization groups despite more pacemaker implantations and drug dose adjustments in the ILR group could indicate that syncope was rarely caused by bradyarrhythmia.^21,22^ Indeed, one could speculate that the ILR monitoring might have resulted in overtreatment with pacemakers. Although only 12% of the many participants with ILR-detected SND ultimately received a pacemaker, 1.8% of all ILR recipients eventually received a pacemaker for this indication, compared with only 0.9% for the control group. High-grade AVB was somewhat more frequently diagnosed in those receiving ILR than in the control group (3.2% vs 1.5%) and also was associated with more pacemaker implantations (2.5% vs 1.5%). A significant proportion of pacemakers were implanted in persons without definite symptoms, especially in the ILR group (Figure 4). The lack of improvement in clinical outcomes supports consideration of symptoms when choosing to implant a pacemaker.

### Bradyarrhythmia Risk Factors

In the control group, the incidence of bradyarrhythmia was approximately 3 times higher than what has been reported in the general population: 0.43 (95% CI, 0.32-0.57) and 1.04 (95% CI, 0.86-1.23) per 100 person-years for female and male persons, respectively, in the control group vs 0.13 (95% CI, 0.12-0.14) and 0.29 (95% CI, 0.27-0.31) for female and male persons, respectively, older than 65 years and participating in the UK Biobank.^23^ The inclusion of more risk-prone participants likely explains the higher rates in the current study, whereas increased awareness on arrhythmias following randomization to usual care may also play a role, keeping in mind that this group was also diagnosed with AF more often than expected.^12^ We confirmed previously identified risk factors for bradyarrhythmia: higher age, male sex, lower resting sinus rate, and history of syncope or coronary artery bypass graft.^23,24,25,26^ Heart failure and valvular heart disease were associated with bradyarrhythmia in the control group only, indicating that the ILR-detected bradyarrhythmias were less likely part of a clinical disease. Interestingly, we found significant interactions between randomization group and age, sex, and history of heart failure or valvular heart disease, indicating that the overdiagnosis of bradyarrhythmias using ILR is more pronounced in persons who are relatively younger, female, and have less structural heart disease.

The high prevalence and overlapping findings of incidental bradyarrhythmias and AF highlights the need to address whether these arrhythmias represent a clinical problem. As previously reported, rhythm or rate control was rarely used, indicating that this coexistence was not attributable to pharmacologic adverse reactions.^27^ Should screening for subclinical AF or bradyarrhythmia prove clinically relevant, a next question will be by which means screening should be performed. Continuous electrocardiographic monitoring will detect low-burden arrhythmias, whereas intermittent or wearable technologies will likely detect more persistent forms.^11^

### Limitations

First, the current study is a post hoc study of a randomized clinical trial with inherent limitations regarding the lack of prespecified outcomes and analyses. Second, the findings rely on data captured from remote monitoring in the ILR group along with study visits and review of health records in both groups, and some bradyarrhythmias or syncope may have been managed without registration in the trial. Arguably, this might have occurred mostly in the control group and mostly with clinically insignificant episodes. On the other hand, whereas the detection rates in the ILR group arguably represent a valid estimate of cumulated subclinical and clinical bradyarrhythmias, the external validity of the control group could be biased toward increased awareness on arrhythmias following randomization, the so-called Hawthorne effect.^28^ Thus, outside the trial, one could anticipate an even larger difference in bradyarrhythmia detection and treatment between screening and no screening. For ILR-detected bradyarrhythmia, the detection was based on R-wave sensing with a very high sensitivity, whereas the specificity was lower, and adjudication relied on best clinical judgment of the physician.^29^

## Conclusions

Bradyarrhythmias were frequently observed in this post hoc analysis of an AF screening trial of persons older than 70 years with cardiovascular risk factors recruited outside the hospital setting. Long-term continuous monitoring led to a 6-fold increase in bradyarrhythmia diagnose and a significant increase in pacemaker implantations compared with usual care, but no change in the risk of syncope or sudden death. The findings indicate that incidentally detected bradyarrhythmia may be a risk marker but is often not a disease itself.

## Notes

Supplement 1. Trial protocol

Supplement 2. eFigure 1. Cumulative incidence of sudden cardiovascular death by randomization arm eTable 1. Concomitant diagnoses of bradyarrhythmia and atrial fibrillation eTable 2. Baseline variables associated with incident bradyarrhythmia

Supplement 3. Data sharing statement
