# Supplementary material for: Prevalence and Prognostic Significance of Bradyarrhythmias in Patients Screened for Atrial Fibrillation vs Usual Care: Post Hoc Analysis of the LOOP Randomized Clinical Trial
Source: JAMA Cardiol. 2023 Feb 15;8(4):326–34. doi: 10.1001/jamacardio.2022.5526 (PMC9932940; doi:10.1001/jamacardio.2022.5526)
Supplement: Supplement 1. — Trial protocol [file jamacardiol-e225526-s001.pdf]

Atrial Fibrillation detected by continuous ECG monitoring using implantable loop recorder to prevent stroke in high-risk individuals -

The LOOP study

By the Steering Committee of the LOOP Study

Trial Sponsor:

The LOOP Study Steering Committee chairman Jesper Hastrup Svendsen
Department of Cardiology, section 2013
Rigshospitalet
Blegdamsvej 9, DK-2100 Copenhagen, Denmark

Protocol Version 63
Version date: 5th July 2019
Approved by the Steering Committee at meeting: August 16th 2019
Protocol number in the Ethics Committe of the Capital Region of Denmark: H-4-2013-025
ClinicalTrials Gov number: NCT02036450

Protocol synopsis

Title:
The LOOP study: Atrial Fibrillation detected by continuous ECG monitoring using implantable Loop recorder to prevent stroke in high-risk individuals.

Background:
Stroke is a major health problem, which affects approximately 16,000 people annually in Denmark, causing severe and burdensome consequences for its victims, who suffer a significant loss in quality of life (QoL), and their families. Furthermore, the healthcare costs due to stroke are sizeable, amounting to more than EUR 38 bn in the EU in 2006. Stroke is often caused by atrial fibrillation (AF), which is the most common type of cardiac arrhythmia. Effective management of AF with anticoagulation therapy is available and may have considerable benefits for patients as well as society. Difficult to detect, however, asymptomatic AF is usually only diagnosed by chance. Recently developed technology (implantable loop recorder; ILR) allows continuous long-term electrocardiography (ECG) monitoring and may constitute a substantial improvement in AF diagnosis. Furthermore, long-term ECG monitoring is essential for studying the significance of AF in stroke pathophysiology. The enormous socio-economic consequences of stroke mean that even a modest reduction in the number of strokes would benefit society.

Primary objective:
The primary objective of this study is to determine whether screening for AF with ILR and initiation of oral anticoagulation (OAC) if AF is detected, will reduce the risk of stroke and systemic arterial embolism in patients with risk factors for stroke.

Secondary objectives:
Secondary objectives include studies of whether the screening will reduce the risk of the combined endpoint of stroke, systemic arterial embolism, and mortality, health economic analyses, quality-of-life assessments, cognitive function assessments, and studies of AF characterization and risk markers from 12-lead ECG, genetics, cardiac and brain imaging, biochemistry, and more.

Study design:
Randomized, un-blinded, controlled parallel two group trial.

Endpoints:
Primary endpoint: Time to first stroke or systemic arterial embolism
Secondary endpoints: Composite endpoint of time to adjudicated stroke, systemic arterial embolism, or death, death (all-cause death, or cardiovascular death), transitory ischaemic attack, intracranial hemorrhage not classified as stroke, major bleeding, atrial fibrillation or flutter, acute (non-elective) hospitalization for cardiovascular disease or complications, requiring an overnight stay in hospital, ischemic events (ischemic stroke or systemic arterial embolism, myocardial infarction, acute limb ischemia, urgent revascularization, or coronary heart disease death), implantation of cardiac pacemaker or defibrillator, ablation of cardiac arrhythmia

Sample size:

In total 6,000 subjects will participate: 1,500 randomized to receive an ILR (ILR group) and 4,500 randomized to receive standard care (control group).

Summary of Subject Eligibility Criteria:

Subjects will be 70 years of age or older and will have at least one of the following diseases: hypertension, diabetes, heart failure, or previous stroke, while exclusion criteria include any history of AF or current contraindication to OAC.

ILR group:

Patients randomized to the ILR group will receive the monitoring device, and if AF is detected, OAC will be initiated according to study guidelines.

Control group:

Patients will be treated according to standard care.

Study Duration:

The study comprises an inclusion period of approximately 2 years followed by at intervention phase of minimum 3 years. The total study duration will therefore be approximately 5 years.

Screening and Randomization:

After randomization, patients allocated to the ILR group should receive the implantation as fast as possible, and preferably within 4 weeks.

Statistical Considerations:

We expect that 30% of the participants will have documented AF episodes during ILR monitoring for at least three years. The study population is expected to have a stroke rate of 0.7%/year in non-AF patients, and 2%/year in AF patients not treated with OAC. We assume that 3% of all participants in the control group will become diagnosed with AF and start OAC. We expect the number of AF-related strokes to be reduced by OAC. The annual rate of the primary end point in the control group is expected to be 1%. Finally, we expect that 5% of the ILR group will not be monitored due to refusal of ILR implantation or ILR explantation due to discomfort. We assume an overall hazard ratio of 0.65 for ILR vs control. To analyse this reduction in the primary endpoint with a 2-sided α-level of 0.05 and a power of 80%, we will need 279 primary events. Follow-up duration will be adjusted to accommodate the number of primary events.

Primary Endpoint Analysis:

The principal analysis for the primary endpoint will employ the intent-to-threat principle and use a survival analysis. To account for the competing risk of death, the cumulative incidences of the primary endpoint will estimated, plotted and group-wise compared in a multi-state fashion. A co-variate adjusted analysis of the combined primary endpoint using a cause-specific Cox proportional regression model will be performed as a supportive analysis. The hazard ratios and the corresponding 95% confidence intervals will be estimated. Subjects completing the study and not reaching the composite endpoint will be censored.

Secondary Endpoint Analysis:

All time-to-event secondary endpoints will be analysed similarly to the primary endpoint.

Event Committee:

An event adjudication committee will classify endpoint events throughout the study.

Funding:

Research grants have been obtained from the Danish National Foundation for Strategic Research (DKK 15.6 mio), the Research Foundation for the Capital Region of Denmark (DKK 2.0 mio) and the Danish Heart Foundation (DKK 150.000). MEDTRONIC Bakken Research Center B.V.(manufacturer of the ILRs will donate an unrestricted research grant to the study covering costs related to 900 ILRs (which has a value of DKK 5.4 mio (reduced price) or DKK 22.5 mio (ordinary list price)) and a grant to cover salary for research nurses at the hospitals (DKK 1.9 mio).

Additional research grants will be applied for from private and public research foundations as well as from industry.

Protocol Signatures:

Responsible for the clinical part of the study:

Professor Jesper Hastrup Svendsen
Department of Cardiology, The Heart Centre, Rigshospitalet
Blegdamsvej 9, 2100 Copenhagen
Phone no. +45 3545 2817; E-mail: Jesper.Hastrup.Svendsen@regionh.dk

Professor Lars Køber
Department of Cardiology, The Heart Centre, Rigshospitalet
Blegdamsvej 9, 2100 Copenhagen
Phone no. +45 3545 3519; E-mail: Lars.Koeber@regionh.dk

Consultant Søren Højberg
Department of Cardiology Y, Bispebjerg Hospital
Bispebjerg Bakke 23, 2400 Copenhagen NV
Phone no. +45 3531 2578; E-mail: soeren.hoejberg@regionh.dk

Consultant Ketil Haugan
Department of Cardiology, Roskilde Hospital
Køgevej 7-13, 4000 Roskilde
Phone no. +45 4732 6000; E-mail: khau@regionsjaelland.dk

Consultant Axel Brandes
Department of Cardiology, Odense University Hospital
Sdr. Boulevard 29, 5000 Odense C
Phone no. +45 6611 3333. E-mail: axel.brandes@ouh.regionsyddanmark.dk

Professor Derk Krieger,
Department of Neurology, Bispebjerg Hospital.
Bispebjerg Bakke 23, 2400 København NV

Responsible for monitoring:

Steering committee:
c/o Professor Jesper Hastrup Svendsen
Department of Cardiology, section 2013
Rigshospitalet, Blegdamsvej 9, 2100 Copenhagen, Denmark

Members of the Steering Committee of the LOOP Study:

Professor Jesper Hastrup Svendsen, Department of Cardiology, Rigshospitalet (chairman).
Professor Lars Køber, Department of Cardiology, Rigshospitalet.
Consultant, associate professor Axel Brandes, Department of Cardiology, Odense University Hospital.
Consultant Ketil Haugan, Department of Cardiology, Roskilde Hospital.
Consultant Søren Højberg, Department of Cardiology, Bispebjerg Hospital.
Professor Derk Krieger, Department of Neurology, Bispebjerg Hospital.
Associate Professor Christian Kronborg, University of Southern Denmark.
Associate Professor Claus Graff, Aalborg University.

Additional Researchers:

Resident Søren Zöga Diederichsen, Department of Cardiology, Rigshospitalet.

Study glossary:

AF: Atrial fibrillation
AE: Adverse event
CIED: Cardiac Implantable Electronic Device
EC: Endpoint Committee
eCRF: Electronic Case Report Form
ILR: Implantable Loop Recorder
QoL: Quality of Life
SAE: Serious adverse event
SC: Steering Committee
Stroke: rapid loss of brain function due to disturbed blood supply to the brain.
TIA: Transient Ischaemic Attack

Table of Contents

Protocol synopsis	2
Protocol signatures	5
Responsible for the study	5
Responsible for monitoring	5
1. OBJECTIVES	9
1.1 Primary	9
1.2 Secondary	9
1.3 Expected results	9
2. BACKGROUND and RATIONALE	10
2.1 Stroke and AF	10
2.2 ECG and AF risk	11
2.3 Health economy and QoL	11
2.4 Study Rationale	12
2.5 Clinical Hypotheses	12
3. EXPERIMENTAL PLAN	12
3.1 Study Design	13
3.2 Number of Centres	13
3.3 Number of Subjects	13
3.4 Recruitment	13
3.5 Estimated Study Duration	13
4. SUBJECT ELIGIBILITY	14
4.1 Inclusion Criteria	14
4.2 Exclusion Criteria	14
5. SUBJECT ENROLMENT	15
5.1 Recruitment and inclusion visit	15
5.2 Subject Registration	16
5.3 Screen Failures	16
5.4 Randomization and Treatment Assignment	16
5.5 Re-Screening	17
6. TREATMENT PROCEDURES	17
6.1 ILR device	17
6.2 ILR procedures	18
6.3 Concomitant Therapy	18
6.4 Prescribed therapy during the study period	18
7. STUDY PROCEDURES	19
7.1 Subject Screening	19
7.2 Follow-up Phase	19
7.3 Endpoint Determination	24
7.4 Genetic analysis and Biomarkers	24
7.5 Strategy for management of low inclusion rate	24
7.6 Study guidelines dealing with definition of AF	24
7.7 Study guidelines how to treat AF detected by ILR	25
7.8 Study guidelines how to treat AF detected by other methods	25
8. REMOVAL AND REPLACEMENT OF SUBJECTS	25
8.1 Removal of Subjects	25
8.2 Replacement of Subjects	26
9. ADVERSE EVENT REPORTING	26
9.1 Definitions	26
9.2 Reporting Procedures for All Adverse Events	27
9.3 Serious Adverse Event Reporting Procedures	27
10. STATISTICAL CONSIDERATIONS	27
10.1 Study Design	27
10.2 Study Endpoints and Subsets	28
10.3 Sample Size Considerations	29

10.4 Interim Analysis 29
10.5 Access to Individual Subject Treatment Assignments 30
10.6 Planned Methods of Analysis 30
10.7 Study Oversight 31
11. INVESTIGATIONAL PRODUCT 31
11.1 Loop recorder 31
11.2 Compliance 31
12. REGULATORY OBLIGATIONS 32
12.1 Ethical considerations 32
12.2 Informed Consent 32
12.3 Independent Ethics Committee 33
12.4 Subject Confidentiality 33
12.5 Investigator Signatory Obligations 34
13. ADMINISTRATIVE AND LEGAL OBLIGATIONS 34
13.1 Protocol Amendments and Study Termination 34
13.2 Study Documentation and Storage 34
13.3 Study Monitoring and Data Collection 35
13.4 Language 36
13.5 Publication Policy 36
13.6 Compensation 37
14. REFERENCES 37
15. APPENDICES 44
Appendix A – Study procedures – summary 45
Appendix B – Composition of Committees 47
Appendix C – Serious Adverse Events 48
Appendix D – Blood Samples for Genetic analysis and Blood Samples for Biomarkers 48
Appendix E – Device information: the Medtronic Reveal® LINQ 49
Appendix F – Definition of composite endpoint 51
Appendix G – Funding 52
Appendix H – Informed consent 53
Appendix I – Guidelines for oral information 54
Appendix J – Endpoint Committee Charter 56

1. OBJECTIVES:

The overall objective of this project is to improve the identification and treatment of patients at risk of stroke as well as identify how significant the contribution from AF is in stroke causation when studied by a technology which identifies all AF episodes including asymptomatic. This will be pursued by three linked studies to be performed simultaneously in a randomized clinical trial where patients at risk of stroke will be divided into two groups, with one group being continuously ECG monitored by an implantable loop recorder (ILR) and the other one not monitored.

Primary objective:

The primary objective of this study is to determine whether screening for AF with ILR and initiation of oral anticoagulation (OAC) if AF is detected, will reduce the risk of stroke and systemic arterial embolism in patients with risk factors for stroke.

Secondary objectives:

Secondary objectives include studies of whether the screening will reduce the risk of the combined endpoint of stroke, systemic arterial embolism, and mortality, studies of bleeding risks, studies of AF characterization and prediction, health economic analyses, quality-of-life assessments, cognitive function assessments, and studies of risk markers from 12-lead ECG, genetics, cardiac and brain imaging, biochemistry, and more.

Expected results:

The LOOP project is expected to provide results in three important areas:

1) It will document whether detection of asymptomatic AF in high risk patients and subsequent anticoagulation therapy will benefit patients in terms of decreased mortality and/or morbidity (AF, stroke, disability etc.), decreased hospitalisation and increased Quality of Life (QoL), not to mention psychological benefits due to being continuously monitored. Potential disadvantages are side effects caused by the ILR and medication. A demonstrated, clinically relevant benefit will have a significant impact on future patient treatment.

2) It will show whether ECG markers can be used as a means to improve identification of patients with asymptomatic AF and this will help predict which patients will develop AF during follow-up. Results will determine if this will allow the risk stratification of patients into several categories and whether patients with the highest risk need closer follow-up than those at lower risk. On a note, the same will be investigated for markers from blood biochemistry, imaging, genetics, and more.

3) It will document via a health economics analysis of whether or not introducing costly monitoring of patients in high risk of stroke events due to AF is cost-effective. The analysis will also show how additional costs and cost savings will be distributed between various sectors and actors. The combined documentation can then be used to inform health policy decision makers.

2. BACKGROUND AND RATIONALE:

Patients with AF, diabetes, hypertension, heart failure as well as previous stroke have increased risk of stroke. Lifestyle factors such as smoking and physical inactivity are also associated with stroke. Furthermore old age is associated with increased risk of stroke. If several risk factors are present in the same patient the risk of stroke is increased further – these individuals are high-risk individuals with respect to stroke.

2.1 Stroke and AF:

Stroke is an increasingly common cardiovascular disease. Out of the 16,000 people suffering annually from stroke in Denmark, 12,000 of them are first-time strokes.[1] More than 85,000 people in Denmark live with a previous stroke.[1] By 2020 120,000 individuals are expected to be living with a previous stroke, which represents a 50% increase from 2000 to 2020.[1] Stroke primarily affects the elderly. Nearly 70% of all strokes appear in people over the age of 65. Worldwide, stroke is the reason for 10% of all deaths and causes 5.7 million deaths annually.[2] The healthcare costs due to strokes in the EU were more than EUR 38 bn in 2006, accounting for 2-3% of total EU healthcare costs.[2]
AF is the most common cardiac arrhythmia. Each year (2009) more than 21,000 people in Denmark experience a first-event AF and more than 64,000 live with an AF diagnosis,[1] the incidence of AF having increased by 35-40% from 2000 to 2009, a development that is expected to continue. Thus the number of new patients with AF is expected to double in 2020 compared with 2000.[1] Other Western countries are experiencing the same type of development.[2,3] AF is associated with having a five times greater risk of stroke.[2,3] Patients with AF-related strokes have a one-year mortality of about 30% and up to 30% of all AF-related stroke victims remain permanently disabled.[2] Thus this patient group has a higher mortality rate and suffers from strokes more disabling than non-AF strokes.[2]
A systematic search for AF primarily by checking for an irregular pulse rate can identify 50-70% more AF patients than a routine strategy based on symptom-driven diagnosis.[3] Thus, putting more effort into detecting AF will mean an increase in AF diagnosis. Previous population-based AF studies have primarily used 12-lead ECG, which only detects a minimum number of cases due to the snap-shot nature of the recording. Correct diagnosis of short-lasting AF episodes is difficult, especially if the episodes are asymptomatic. Continuous long-term ECG monitoring appears to be the ideal method to identify the true incidence of AF and to characterise its subtypes. One technology which can be used for continuous long-term monitoring is a pacemaker. This technology was used in the ASSERT Study, which recently showed that among 2,580 pacemaker patients over the age of 65 with hypertension and without a history of AF, 261 (10.1%) had device-detected asymptomatic atrial tachycardia (mostly AF) at the three-month follow-up.[4] After 2.5 years of follow-up, 51 strokes (or embolic episodes) were detected, 11 of which were detected among 261 (4.2%) of the pacemaker patients with asymptomatic AF while 40 were among the remaining 2319 (1.7%) patients without asymptomatic AF. None of the stroke patients had symptomatic AF at the three-month follow-up.[4]

A less invasive technology which can be used for continuous long-term monitoring is the implantable ILR. This device, which measures 45 mm x 7 mm x 4 mm and weighs 2.5 g, is implanted subcutaneously on the chest wall without the need for electrodes in the heart chambers. The device samples ECG information automatically, based on specific criteria, or on-demand if the patient activates data sampling due to symptoms. Currently, ILR technology provides the best possibilities for continuous monitoring of the heart rhythm for AF detection. The fact that follow-up can be performed by telemedicine is also advantageous.
Anticoagulation therapy is effective in reducing stroke incidence in patients with AF. However, at present we do not have clear evidence about how accidentally detected AF should be weighted with respect to anticoagulation therapy.

2.2 ECG and AF risk:

Most elderly patients who develop AF have structural and functional abnormalities in the atrial myocardium.[5,6] These changes may cause heterogeneous atrial activation and a shortening of the atrial refractory period, resulting in changing patterns of cardiac impulse propagation.[7] The Framingham Heart Study showed that prolongation of the electrocardiographic PR interval is associated with an increased risk of future development of AF.[8] Our research group has recently shown that both an increment of the PR interval and an increment in QTc interval are associated with an increased risk of AF, suggesting that the PR interval, QTc interval and AF have a shared pathophysiology. The atrial repolarisation, and more specifically the atrial effective refractory period, is probably the most important factor in the pathophysiology of AF. The ventricular repolarisation time, reflected in the QT interval, is expected to be a marker of the atrial repolarisation time, as the atria and ventricles share many of the same ion channel complexes. Other ECG markers, such as P-wave duration and morphology, and premature atrial and ventricular contractions, are also associated with future development of AF.[9] This evidence suggests that future AF can be predicted from ECG markers measured on a single lead of the standard electrocardiogram.

2.3 Health economy and QoL:

The direct costs of stroke including acute care and rehabilitation are substantial. Previous studies from Denmark show that the mean cost of healthcare and social services during the first year after stroke was DKK 193,400 per person (2011 price index),[10] and that the initial severity of the stroke was the only medical factor that influenced length of hospital stays.[11] In addition to the cost of stroke treatment, rehabilitation and long term care, stroke diagnosis is associated with a significant loss of QoL for the patient.[12] Thus, interventions that prevent stroke may lead to significant cost savings that may counter balance (some of) the costs of the intervention and also improve the QoL of patients.
Several studies have explored the cost-effectiveness of various stroke rehabilitation strategies[13] and a number of studies have looked at the cost-effectiveness of secondary prevention of stroke with anticoagulation therapy in patients with AF.[14,15,16]

2.4 Study Rationale:

Patients at risk of stroke due to age, or presence of specific diseases may also have undetected atrial fibrillation. If atrial fibrillation without symptoms is detected by continuous ECG-monitoring by a Loop Recorder, this may detect atrial fibrillation, that would not otherwise be detected, and thus the patient gets a possibility to receive anticoagulation therapy, which potentially may reduce the risk of future stroke. However, anticoagulation therapy may also have undesired side effects. Therefore, we will perform a study, where we will record both the beneficial and harmful effect in a randomized trial, and we will assess economical and quality of life aspects in a randomized trial.

2.5 Clinical Hypotheses:

2.5.1 Major hypothesis:

When AF is detected and relevant anticoagulation therapy is initiated in high-risk patients, a reduction in strokes will follow. Similarly, death rate and acute hospitalisations will decrease (secondary end-points).

2.5.2 Secondary hypotheses:

a. Markers from ECG, blood biochemistry, imaging, genetics, and more, can be used to predict AF.
b. Continuous ECG monitoring with an ILR to detect asymptomatic AF and subsequent anticoagulation therapy is cost effective (ie. the cost of monitoring and therapy can be balanced against reduced costs of stroke treatment and rehabilitation, as well as improved QoL and survival).
c. Continuous ECG monitoring can detect other (non-AF) arrhythmias requiring therapy in patients at risk of stroke.

2.5.3 Supplementary hypotheses:

a. Cerebral abnormalities including magnetic resonance imaging (MRI) detected white matter hyperintensity and brain infarcts (at inclusion) are associated with AF and recurrent stroke.
b. Genetic variations are associated with development of AF and certain genetic patterns will prove to be protective against AF.
c. AF burden can predict development of cognitive dysfunction.

3. EXPERIMENTAL PLAN:

3.1 Study Design:

This is a prospective randomized, open-label (unblinded), controlled, parallel group multi-center study performed in Denmark. Participants are recruited following identification from Danish registries as well as from outpatient clinics, and after hospitalizations. The study will recruit individuals residing in Region Zealand, Region of Southern Denmark or the Capital Region of Denmark. Potentially, the study region will be increased to cover other parts of Denmark. Participants are randomized to either receive an ILR (ILR group) or not receive one (control group). The randomization occurs at a ratio of 1:3 (ILR group vs. control group). The LOOP study will comply with the standards of the CONSORT Statement. [17]

After successfully completing all screening and baseline procedures, subjects will be randomized in a ratio of 1 to 3 to receive an ILR or usual control. The ILR will be programmed according to study recommendations (see section 6.2).
The study flow chart is given in Appendix A and the study endpoints are defined in section 10.2.
A steering committee (SC) will oversee the conduct of the study. Composition and roles of this and other committees are described in section 10.7.

3.2 Number of Centres:

The study will be a multi-centre study performed in the south and eastern part of Denmark, where all hospitals involved with implantations of cardiac implantable electronic devices (CIEDs) will be invited to participate. All participating centres will be involved in randomization, implantation of the ILRs as well as follow-up.

3.3 Number of Study Subjects:

Participants in this clinical investigation shall be referred to as subjects or patients. Approximately 6,000 subjects will be randomized in a ratio of 1:3 to receive an ILR (ILR group) or continue standard care regimen (control group).
It is expected, that the randomization period will last for 2 years. The decline rate is estimated to be <10 %. The cross over from control to the ILR group is expected to be <10 %, since an ILR implant is generally assumed to require a syncope or definite clinical suspicion of arrhythmia or a wish to quantify arrhythmia burden. Subjects discontinuing from the study prematurely will remain in endpoint follow-up unless consent is withdrawn. Statistical considerations are provided in section 10.3.

3.4 Recruitment:

Invitation letters will be sent to approximately 15,000 individuals from the general population, identified from registers. We expect that 50% (7,500) will be interested in participating, and of these 20% (1,500) are not expected to be able to participate due to the exclusion criteria. Thus, we expect to include the planned 6,000 individuals. At each of the four hospitals 1,500 patients will be randomized (based on the assumption of 3-4 patients per working-day and 200 working-days per year).

3.5 Estimated Study Duration

The study is expected to conclude after 3 years of follow-up. The SC will continuously monitor the overall event rate and will recommend if the follow-up period should be extended, based on the number of observed primary endpoints. Based on the event rate the SC may also recommend to alter the sample size in order to complete the trial in approximately 5 years from the start of the recruitment phase.

4. SUBJECT ELIGIBILITY:

The investigators will maintain a screening log, that will include limited information about all identified potential study candidates (e.g., initials, age, sex, race, date) and outcome of the screening process (e.g. enrolled into study, ineligibility (and reason for ineligibility), or refusal to participate).

4.1 Inclusion Criteria:

To be eligible for the study, subjects must fulfil the following criteria at inclusion:

- Living address in Region Zealand, Region of Southern Denmark or the Capital Region of Denmark (study subjects from other regions of Denmark who actively contact the study for participation can be included if they wish to participate)
- 70-90 years of age at the time of screening

and additionally have at least one of the diseases mentioned below:

- Diabetes mellitus (type 1 or type 2, with or without medical therapy)
- Hypertension (with or without a therapy that may reduce blood pressure; i.e., an increased blood pressure at randomization is not required)
- Congestive heart failure
- Previous stroke (preferably diagnosed in hospital and verified by imaging; previous TIA is not considered an inclusion criterion)

Concerning the four qualifying diseases, the study subject is considered qualified for study inclusion if he has a history of one of the diseases (for example subject is now normotensive on life-style correction or medical therapy and similarly if blood glucose has been normalised upon life-style corrections or medical therapy).

4.2 Exclusion Criteria:

To be eligible for this study subjects must not meet any of the following criteria:

- History of atrial fibrillation or flutter irrespective of type
- Cardiac pacemaker or defibrillator (with or without re-synchronization therapy)
- Contraindication to oral anticoagulation therapy
- Anticoagulation therapy; vitamin K antagonists, direct oral anticoagulants, or (low-molecular) heparins. Therapy with platelet inhibitors such as acetyl-salicylic acid, clopidogrel, persantine is not considered an exclusion criterion
- Renal failure treated with permanent dialysis
- Uncorrected congenital heart disease, or severe valvular stenosis, obstructive cardiomyopathy, active myocarditis, or constrictive pericarditis.
- On a waiting list for major surgery (cardiac, thoracic or abdominal)
- Cardiac or thoracic surgery has been performed within 3 months from inclusion

- Any major organ transplant (e.g. lung, liver, heart, or kidney)
- Cytotoxic or cytostatic chemotherapy and/or radiation therapy for treatment of a malignancy within 6 months before randomization or clinical evidence of current malignancy with the following exceptions: Basal or squamous cell carcinoma of the skin, cervical intraepithelial neoplasia, prostate cancer (if stable, localized disease with a life expectancy of > 2.5 years in the opinion of the investigator)
- Life-expectancy shorter than 6 months
- Known to be human immunodeficiency virus (HIV) positive with an expected survival of less than 5 years due to HIV infection
- Recent (within 3 months) history of alcohol or drug abuse based on self-reporting
- Any condition (e.g. psychiatric illness, dementia) or situation, that in the investigators opinion could put the subject at significant risk, confound the study results, or interfere significantly with the subject participation in the study
- Unwillingness to participate or patient does not understand Danish language

Study subjects who during follow-up develop one of the mentioned diseases which qualify as an exclusion criterion at baseline will remain in the study and complete the follow-up.

5. SUBJECT ENROLMENT:

5.1 Recruitment and inclusion visit

Potential study subjects will be contacted by an invitation letter about the LOOP study with initial basic information about the study. If interested in participating in the study, potential study subjects are asked to contact one of the participating hospitals (typically the geographically closest) and an out-patient consultation is scheduled (inclusion visit).

At the inclusion visit the study subjects will be informed about the study by an investigator (or assigned designee), who will also look up the subject's medical records, and will interview the patient with a focus on previous medical history (diabetes, heart disease, hypertension, AF etc.) to assess study eligibility.

Blood pressure will be measured, and an ECG and blood sample is taken. Baseline variables including CHA_2DS_2-VASc and NYHA class score will be recorded. Individuals with AF on the ECG will be excluded from participating and these patients will be referred for relevant treatment. Subjects will be screened for previously unknown diabetes and hypertension. If these diseases are detected the patients will be referred for further tests or therapy. Individuals who wish to participate will sign informed consent documents, complete the QoL questionnaires and The Montreal Cognitive Assessment (MoCA) tests, and will be randomized to either implantation of ILR or standard care. Subjects must personally sign and date the consent form.

Patients may have additional study tests performed (T-wave alternans test, echocardiography, MRI etc.).

For the purpose of this trial the following definitions will apply:

Registration

A subject has signed the informed consent form and has been assigned a subject identification number generated by the computer generating code (but not yet randomized)

Randomization

A subject has been assigned to one of the two treatments and has been assigned a randomization number.

Enrolment

A subject is considered enrolled once the subject is randomized and the ILR implantation (or control) has been performed.

5.2 Subject Registration:

After the provision of informed consent each subject will be registered in a web-based electronic eCRF (eCRF), protected by encryption. A subject identification number will be assigned during registration, and will be used to identify a subject throughout the trial and must be used on all study documentations related to that subject. The subject identification number must remain constant throughout the entire trial; it must not be changed at any time.

All entries in the eCRF up to and including randomization are performed at the participating centre. Subsequent follow-up visits can be entered at the participating hospital where the patient is being followed. Subject initials and date of birth as well as other information will be required for registration, including for example subject initials and date of signed informed consent.

5.3 Screen Failures:

Registered subjects, who are ineligible for the study based on screening assessments will be considered screen failures and entered as such into the eCRF. The following information will be required:

- Subject identification number
- Date of birth
- Reason for failing screening
- Date of last screening assessment

5.4 Randomization and Treatment Assignment

Subjects, who successfully complete the screening assessment, and meet the eligibility criteria, will be randomized into the study. A computer generated randomization scheme will be generated to assign subjects to treatment, and those who fulfil the eligibility criteria (section 4) will be randomized in a ratio

of 1:9 to receive an ILR or to be a control, respectively. The investigator (or assigned designee) will access the central computer and obtain an assignment code at the randomization visit. The following information at a minimum will be required for randomization:

- Subject identification number
- Date of birth
- Confirmation, that the subject meets the eligibility criteria

This information will be entered into the eCRF. Subject must be randomized within 30 days of being identified at the referral hospital.

5.5 Re-Screening:

Registered subjects, who do not meet all eligibility criteria on the first screening occasion may be rescreened for the study at a later time. Such subjects may begin screening procedures again two weeks after the date of the last screening assessment. Rescreened subjects will retain the originally assigned subject identification number: However, the rescreening attempt will need to be entered into the registration computer.

6. TREATMENT PROCEDURES:

Implantation of a Loop Recorder (ILR) will be performed according to local standards and within four weeks of randomization.

6.1 ILR device:

Devices from Medtronic (Reveal LINQ or newer comparable devices) with capability for remote monitoring will be used in this trial. Information on specific ILR data can be found in Appendix F.

6.2 ILR procedures:

6.2.1 Preparations and setting:

Preoperative biochemical parameters as required by local guidelines (which may include variables such as INR, blood cells, creatinine, sodium, potassium).

The device is implanted at the hospital using standard sterile technique; not necessarily in an operation or cardiac catheterization facility. An intravenous injection line (such as Venflon) is optional, but is inserted if i.v. antibiotics are given.

6.2.2 Medication in relation to surgery:

Antibiotics are given according to study guidelines. It is suggested to give at least one dose of i.v. cefuroxime (Zinacef) 1.5 g or dicloxacillin (Diclocil) 0.5-1.0 g immediately prior to the procedure, or oral dicloxacilling (Dicillin) 0.5-1.0 g at least one hour prior to the procedure. It is optional to give additional antibiotics. In case of allergy to penicillin alternative antibiotics is recommended according to local

guidelines. Sedatives are not recommended. Local anaesthesia is given according to each centre's local guidelines.

6.2.3 The surgical procedure:

The ILR is positioned subcutaneously on the left side of the chest using supplied incision and insertion tools. Measurement of the device orientation is optional. Securing optimal electrocardiogram before wound closure is recommended. At each centre, the implantation of the ILR is performed by either a senior cardiologist or a medical doctor under general supervision of the local senior cardiologist.

6.2.4 Loop Recorder programming:

The ILRs should be programmed according to study guidelines, and details are specified in Appendix E.

6.2.5 Post operative monitoring:

After the implantation, one hour of observation at the facility is recommended , and after this the patient can be discharged from hospital. The patients will receive advice with respect to skin inspection in the surgical region, treatment of local pain after surgery.

6.2.6 Remote follow-up:

Study participants will be instructed in the automated daily remote transmissions which require bedside setup of the MyCareLink Patient Monitor at their home along with patient-initiated transmissions to transfer extra data .

6.3 Concomitant Therapy:

Throughout the study investigators may prescribe any concomitant medications or treatments necessary to provide adequate supportive care. Information on concomitant therapy will be collected on the appropriate case report form.

If the ILR monitoring detects severe bradycardia, ventricular tachyarrhythmia or other arrhythmia these will be dealt with according to clinical guidelines. For study guidelines how to treat AF, see section 7.7. It is possible for the patient to participate in another trial, both including devices (such as stents) or medical therapy.

6.4 Prescribed therapy during the study period:

All subjects (both ILR group and control patients) should receive optimal medical therapy for their inclusion criteria disease (hypertension, diabetes, heart failure or stroke). The national guidelines for treatment of heart failure and hypertension are described in the guidelines from the Danish Society of Cardiology which can be downloaded from the web page www.cardio.dk. The standard regimen for stroke patients is described in the Danish Reference Programme 2013 (Dansk Apoplexi Selskab).

7. STUDY PROCEDURES:

A tabular schedule of study procedures is given in Appendix A. Written informed consent must be obtained before performing screening procedures. The investigator is responsible for ensuring that all assessments are performed according to the protocol and that the appropriate data are recorded in the eCRFs. Missed visits, test(s), that are not done and examinations, that are not conducted, must be reported on the eCRFs.

7.1 Subject Screening:

During screening each subject will be evaluated for inclusion in the study. All laboratory assessments scheduled for screening will be done by the local laboratory. The following procedures, screening evaluations and assessments will be done once within 21 days prior to randomization:

- Obtaining or verifying informed consent
- Medical history, including previous, surgical procedures
- NYHA functional classification: The severity of heart failure will be evaluated by the investigator or the designee at the respective study visits. If possible a subject should be evaluated by the same person throughout the study.
- CHADS-VASc score
- Physical examination, including height, body weight
- Ongoing medical therapy
- Blood samples for routine biochemical testing, plasma troponines, p-BNP and for storage in the study related research biobank. After analysis of the material from the study related research biobank surplus biological material is transferred to a biobank for future research.
- 12-lead ECG

If laboratory samples are missing, destroyed, unable to be analysed or results are considered and documented by the investigator as being obviously incorrect, a subject may be re-tested.

7.2 Follow-up Phase:

7.2.1. The main study:

The ILR devices will be implanted over the course of 12-24 months (1-8 implantations per week in each of the centres), starting at the beginning of 2014.

- Participants in the ILR group will be seen at in-hospital visits at 12, 24 and 36 months after ILR implantation. Between in-hospital visits they will be followed by daily automatic ILR remote telemedicine transmissions. Data from the ILRs are automatically transmitted to a central database. Every day, an experienced medical doctor reviews the recent transmissions in the database. Subjects will be contacted if transmissions are not received in a period of 14 days, and any system malfunctions will be corrected. If a new-onset AF episode lasting minimum 6

minutes is detected, two senior cardiologists independently evaluate the transmission. In case of disagreement, a third senior cardiologist will independently evaluate the episode. If the two or two of three cardiologists confirm the diagnosis of AF, OAC will be initiated by the local center (see section 7.8), and this will be registered in the eCRF. Remote monitoring will continue until the end of battery life, study withdrawal, or death occurs. At in-hospital visits, QoL and MoCA forms will be filled. Adverse events will be collected at study visits and by lookup in medical records and registries at least on a yearly basis until the end of study.

- Participants in the control group will be seen only at inclusion and after the 3 year follow-up. During this period they will be seen by their general practitioner. Between the scheduled visits QoL forms will be sent by mail. MoCA forms will be filled in on the final follow-up. Adverse events will be collected at study visits and by lookup in medical records and registries at least on a yearly basis until the end of study.

Additional visits are made at the discretion of the caring physician. Throughout the study the investigator or study nurse/coordinator should continue to provide appropriate medical care.

The following assessments and procedures will be performed at specified time points during the study and will be recorded in the eCRFs (for details please see Appendix A, section a and b):
- CHADS-VASc score: At randomization.
- Blood sample: At randomization. Some participants might have additional blood samples taken.
- NYHA functional classifications: At randomization and (12, 24,) 36months.
- Assessment of QoL and cognitive function : At randomization, months 12, 24 and 36.
- Vital signs (BP, HR …): At randomization and at (12, 24), 36 months.
- 12-lead ECG: At randomization and at (12, 24), 36 months.
- Concomitant therapy: At randomization and months 12, 24, 36 and by lookup in medical records and registries at least on a yearly basis.
- Remote Loop Recorder readings: Daily
- Adverse events (AEs): At clinical visits and by lookup in medical records and registries at least on a yearly basis (see section 9 for definitions).

Information on all AEs, that occur from study day 1 after randomization will be recorded in the appropriate eCRFs on study visits and by lookup in medical records and registries at least on a yearly basis. All serious adverse events (SAEs) will be reported to the study office within one working day of discovery (see appendix C), and a SAE form must be completed (see section 9.3 for definitions). Bleeding complications will be registered according to the suggestions by the International Society for Thrombosis and Haemostasis (Schulman and Kearon, 2005). For the efficacy analysis of mortality, the endpoint committee (EC) will review all deaths and classify the events as cardiovascular or non-cardiovascular (see Appendix J). Cardiovascular deaths will be further classified as sudden and non-

sudden based on a time criteria (see Appendix J). Adjudicated cause of death as well investigator-reported cause of death will be entered into the eCRF.

A subgroup of those patients that underwent an echocardiography at inclusion will be invited to participate in a similar echocardiography during follow-up. The criteria for invitation is that the participants should have been included in the LOOP study before January 2017 at one of the study centres Rigshospitalet, Bispebjerg Hospital or Roskilde Hospital, and that the first echocardiography should be between 2 and 6 years old at the time of follow-up echocardiography.

For endpoints, see section 10.2

7.2.1 The ECG study:

The ECG computer analysis study will collect periodic data from the ILR during sinus rhythm and during episodes of AF (both patient-activated and automatic sampling). Patients in the ILR group will be encouraged to make a monthly patient-activated sampling after five minutes of rest (most likely in sinus rhythm). Standard 12-lead ECGs will be obtained at baseline (ILR and control groups) and also annually in the ILR group. ECG markers predictive of future AF will be derived from the recordings.

<u>Objectives:</u>

Primary: To study the the association between chronological developments of ECG markers measured by ILRs and incident AF, in order to identify which patients fall into low or high risk AF groups.

Secondary: Calculation of daily AF burden (ILR group) to refine quantification of episodes beyond merely presence/absence and to guide decisions regarding the need for anticoagulation. Relationship between AF burden and development of ECG markers.

Additional ECG studies:

- Incidence rate and timing of new onset AF to identify the necessary duration and regularity of ECG monitoring to reliably identify change in status.
- Throughout the ECG study we will also monitor the frequency of symptom triggered ILR transmissions, the proportion of falsely detected AF episodes by ILR as well as the number of planned ECG samplings and data transmissions from ILR devices which are not received.
- Bradyarrhythmia and ventricular tachyarrhytmia.

7.2.2 The health economic study:

The health economic analysis is appended to the analysis of the randomized clinical trial.

Objectives:

Analysis of changes in the use of healthcare resources and costs, i.e. hospital treatment, general practitioner services, medical specialists, pharmaceuticals, rehabilitation, and nursing homes, and analysis of quality-adjusted life-years (QALYs).

Comments:

Individual personal data on resource use and health outcomes will be collected prospectively during the trial or from national registers, which also continuously record healthcare consumption. The framework for the analysis is a cost-utility analysis where the mean differences in costs and QALYs between the intervention and the control group is expressed as a ratio that shows the cost per QALY gained from the introduction of ILR monitoring and subsequently AF management. All costs will be estimated from a societal perspective.

Resource use data in relation to the implantation of ILR, ILR data transmission and analysis, as well as follow-up visits, will be recorded prospectively. The registration will include noting the amount of time staff spend during pre-operative evaluation, procedures (e.g. blood tests, electrocardiogram), utensils, anaesthetics, sedatives, antibiotics and hospital stay for each participant. In order to estimate costs, information about salaries, the cost of utensils, pharmaceuticals and equipment will be collected from the hospital finance department.

Data on the participants' use of inpatient and outpatient hospital care and use of emergency departments, general practitioners and other private practising medical specialists, physiotherapists, chiropractors, chiropodists and psychological counsellors, as well as prescription drugs, will be collected from the National Patient Register, the National Insurance Service Register, and the Register of Medicinal Product Statistics. It is assumed that costs of healthcare consumption unaffected by the intervention will cancel out due to randomization. Furthermore, it is assumed that all AF management costs will be captured in the register data.

Hospital treatment will be valued with official hospital charges (diagnosis related group (DRG) charges) provided by the National Board of Health. Special attention to the relevance of using DRG charges will be considered.[21] Services from private practising providers will be valued according to the prevailing fee schedules that are agreed upon between the providers and the regional health authorities. For the valuation of general practitioner services an annual amount will be added to take into account that general practitioners are paid by a combination of capitation and fee-for-service. Furthermore, patients' co-payments will be added. Data on the participants' use of prescription drugs will be collected from the Register of Medicinal Product Statistics. These drugs will be valued according to the pharmacy sales prices.

Information about the participants' use of services provided by the municipalities, e.g. community nurse visits, personal care and practical assistance at home, nursing home, respite care and rehabilitation, will be collected from the participants' municipality of residence. Unit costs of these

services will be estimated based on statistics on wage rates and databases that record the cost of providing home care, (e.g. The Free Choice Database (*Fritvalgsdatabasen*)), which is administered by the National Board of Social Services. All costs will be converted to a specific year price level using the consumer price index and other relevant price indices.

QALYs will be estimated with the use of the EQ-5D-5L instrument.[12] Participants will fill in the EQ-5D-5L questionnaire at baseline (before operation) and every second year (mailed to control group). Each health state is converted to a social tariff (utility score) from a representative sample of the Danish population.[22] This will be used to construct each participant's utility profile. A straight line relation between each of the observed utility scores will be assumed. The number of QALYs experienced by each participant will be calculated as the area under the participant's utility profile.

Descriptive analyses will be used to describe the mean QALYs, resource use and costs per participant in both arms of the trial. Differences in QALYs, resource use and costs between the two groups after 24 months of the study will be based on the trial data. Confidence intervals of 95% for differences between the two groups will be computed based on bootstrap re-sampling with 5,000 replications of the trial data.

For the cost-effectiveness analyses, the incremental cost-effectiveness ration (ICER) will be estimated on the basis of the mean difference in costs relative to the mean difference in QALYs. Uncertainty about the ICERs will be analysed using bootstrapping and presented in a cost-effectiveness plane. A cost-effectiveness acceptability curve will be generated to represent the probability that ILRs are cost-effective compared with usual treatment. Estimates of the costs per QALY gained from ILR will be compared to similar estimates of rehabilitation after stroke, AF management as well as other cost-effectiveness estimates in other treatment areas.

Study nurses will register all contacts between study subject and study nurse in the hospital files to be included in the cost evaluation.

7.2.3 The MRI study:

Patients with risk factors for stroke frequently have asymptomatic previous brain infarcts.[19] Age, hypertension and diabetes predispose to white matter hyperintensities, which is associated with cognitive disorders, balance and mood disorders.[20] In a subset of the study participants (approximately 500 subjects) we will perform MRI of the brain upon study entry to detect previous brain infarcts (semi-quantitative assessment) and white matter hyperintensities (semi-quantitative assessment). MRI scans will be performed without contrast injection. In the patients in whom stroke occurs during follow-up a second MRI should be performed close to the time of stroke. In the subjects who had a first MRI scan performed a final MRI should be performed at the end of the study. MRI scans of brain are performed with 3 sequences: FLAIR , DWI and Gradient Echo sequencing. In a subset of participants we will perform an MRI scan of the heart with Gadolinium contrast to measure chamber dimensions, wall-motion and late enhancement (fibrosis) (approximately 200 subjects).

7.3 Endpoint Determination:

7.3.1 Definition of Endpoints:

Please see Section 10. Details are provided in Appendix F.

7.3.2 Data Collection:

Endpoint data will be collected on specific eCRFs. For all possible primary endpoints, and all deaths, all supporting documentation from medical records, imaging, laboratory tests, registries, etc., will be collected, and will be provided to the EC during the course of the trial for review and confirmation of events as outlined in the EC charter.

7.4 Genetic analysis and Biomarkers:

At randomization 3 types of blood vials will be collected (whole blood sample, EDTA while sample, and serum sample). Please see Appendix D for handling of blood samples. Blood samples will be used for genetic and biochemical analyses.

Other markers will be acquired as described above: ECG, and MoCA test (and imaging in a subset of patients) .

7.5 Strategy for management of low inclusion rate:

1) Too low patient recruitment due to healthcare professionals or logistics: We will continuously monitor patient inclusion each month per centre. If a centre does not recruit the expected number of patients we will recruit a new centre. In the interim period the other centres will increase their recruitment.

2) Too low patient recruitment due to patient preferences: We conducted a small survey asking potential candidates if they would participate if asked, and patients were positive. A logbook will be used to list and identify reasons why patients decline to participate. If better information can increase the participation rate we will improve the quality of the information. Otherwise additional letters of invitation will be sent.

3) Patients cannot perform telemedicine transmissions: Rigshospitalet is currently performing another study with ILRs in elderly patients with valve disease (aortic stenosis). These patients are fully capable of performing telemedicine transmissions. In the event of problems, study nurses will guide the patients by telephone.

7.6 Study guidelines dealing with definition of AF:

Arrhythmia which the device has sampled and categorised as AF and which is afterwards:

- confirmed as AF by a study investigator and
- has a duration of ≥ 2 minutes (please note that this is not the same as AF which requires anticoagulation therapy)

After new AF has been diagnosed the patient will continue unaltered arrhythmia monitoring as previously performed. The device requires 2 minutes for AF detection.

7.7 Study guidelines how to treat AF detected by ILR:

When AF is detected by the ILR and the episode is verified by an investigator and has a qualifying duration (i.e. an episode which has a duration ≥ 6 minutes), the patient is given advice to start therapy with one of the newer OAC (preferred strategy) or conventional OAC according to local routine.

It is recorded if the patient actually will receive OAC. Subjects will be covered by normal patient insurance.

7.8 Study guidelines how to treat AF detected by other methods (also relevant for the control group):

When AF is diagnosed in the control group or in the ILR group by non-ILR methods (including monitoring during hospitalisations, Holter, ECG, etc) patients are recommended to start anticoagulation according to the same guidelines as mentioned in the section above (section 7.8).

8. REMOVAL AND REPLACEMENT OF SUBJECTS:

8.1 Removal of Subjects:

A subject has the right to withdraw from the study at any time, for any reason, without prejudice to his or her future medical care by the physician or at the institution. Any subjects who withdraws consent to participate in the study will immediately be removed from further treatment and/or study observations on the date of request. The investigator has the right to withdraw the subject from the study, if any of the following occurs:

- Significant illness caused by the ILR
- Refusal by the subject to continue observations
- Decision by the investigator, that termination is in the subjects best medical interest
- Loss to follow-up

Should a subject (or legally acceptable representative) request or decide to withdraw from the study, all efforts will be made to complete and report the observations as thoroughly as possible up to the date of withdrawal. All information should be reported on the applicable eCRF. A complete, final evaluation and assessment (section 7.4) should be made at the time of the subject’s withdrawal. The end of study eCRF will be completed with an explanation for the withdrawal. If the withdrawal of a subject is due to an AE, follow-up visits should be scheduled until the AE has resolved or stabilized. Unless consent has been withdrawn, follow-up date on death and hospitalizations will be collected until study termination, though if specifically requested by the patient, the patient will not be followed for the primary endpoint until end of study.

8.2 Replacement of Subjects:

Randomized subjects will not be replaced if discontinued.

9. ADVERSE EVENT REPORTING:

9.1 Definitions:

9.1.1 Adverse Events:

An AE is defined as stroke (hemorrhagic or ischemic), peripheral emboli, bleeding, cardiac event (atrial fibrillation, ventricular or supraventricular tachyarrhythmias, bradycardia, pacemaker implantation, myocardial infarction, unstable angina, percutaneous coronary intervention, coronary artery bypass graft or heart failure) or ILR infection, occurring in a subject after the initiation of the study, whether or not considered to be related to the implantation of the ILR. Elective hospitalizations (i.e., due to any pre-existing systemic illness or elective surgery) are not AEs. Abnormal laboratory values should not be reported as AEs, unless they fulfil the definition above.

9.1.2 Serious Adverse Events:

A serious adverse event is defined by regulatory agencies as an AE that poses a significant hazard or side effect, regardless of the investigators or sponsors opinion on its relationship to the investigational product. This includes, but may not be limited to, any event, that:

- Is fatal, or
- Is life threatening (places the subject at immediate risk of death) , or
- Requires in-patient hospitalization or prolongation of existing hospitalization, or
- Leads to persistent or significant disability/incapacity

A hospitalization meeting the regulatory requirement for “serious” criteria is any in-patient hospital admission, which includes a minimum of an overnight stay in a health care facility. Any event, that does not exactly meet this definition, which in the investigators opinion represents a significant hazard, can be assigned “other significant hazard” regulatory reporting serious criteria. Additionally, important medical events that may not be immediately life-threatening or result in death or hospitalization, but which may jeopardize a subject or require intervention to prevent the outcomes listed above, or result in urgent investigation, may be considered serious. Examples include allergic bronchospasm, convulsions or blood dyscrasia. Serious Adverse Events will be reported based on patients information to study personal or monthly registry retrievals and will be filed in CRFs by the local study team.

9.2 Reporting Procedures for All Adverse Events:

All AEs occurring after randomization observed by the investigator or reported by the subject (whether or not attributed to the investigational product), will be reported on the electronic case report form. The following attributes must be assigned by the investigator:

- Diagnosis.
- Description: E.g. hospital records.
- Dates of onset and resolution and action taking.

The investigator may be asked to provide follow-up information. All AEs, serious or not, that result in a subjects permanent withdrawal from the study must be reported in the CRF. The end-of-study eCRF will be completed, giving details of the withdrawal.
Medically significant AEs considered related to the investigational product by the investigator or the sponsor will be followed until resolved or considered stable.

All deaths occurring in study must be recorded, and cause of death must be investigated. This includes deaths until determination of the study or a maximum of 60 months since enrolment of the subject, whichever is earlier.

It will be left to an investigators clinical judgement as to whether or not an AE is of sufficient severity to require a subjects removal from treatment.

9.3 Serious Adverse Event Reporting Procedures:
All SAEs must be reported in the electronic CRF within one working day of discovery or notification of the event. Initial SAE information and all amendments or additions must be recorded on a Serious Adverse Event Report form.
Description of SAEs reporting is included in Appendix C.

10. STATISTICAL CONSIDERATIONS:

10.1 Study Design:
This is a prospective randomized, open-label (unblinded), controlled, parallel group multi-center study performed in Denmark. Patients are recruited following identification from Danish registries as well as from outpatient clinics, and after hospitalizations. The study will recruit individuals who live in the Capital Region of Denmark, Region Zealand or the Southern Region of Denmark. The study is expected to conclude when 279 strokes have occurred or 6,000 study subjects have been followed for at least 36 months, which is expected to be approximately 5 years after enrolment of the first subject. Subjects meeting the eligibility criteria will be randomized to either receive an ILR (ILR group) or not ie, continue standard care (control group). The randomization occurs at a ratio of 1:3 (ILR group vs.

control group) as described in section 5.2. The LOOP study will comply with the standards of the CONSORT Statement.[17] The study design is further described in section 3.

10.2 Study Endpoints and Subsets:

10.2.1 Primary Endpoint:

Efficacy (time to at least one of the components of the combined primary endpoint):

- adjudicated stroke or
- adjudicated systemic arterial embolism

10.2.2 Secondary Endpoints:

- Composite endpoint of time to adjudicated stroke, or systemic arterial embolism, or cardiovascular death
- Time to death by any cause
- Time to cardiovascular death
- Time to adjudicated ischemic stroke
- Time to adjudicated transitory ischaemic attack
- Time to diagnosis of atrial fibrillation
- Time to diagnosis of atrial flutter

10.2.3 Other Endpoints:

- Time to adjudicated intracranial hemorrhage not classified as stroke
- Time to major bleeding as defined by the International Society on Thrombosis and Haemostasis criteria
- Time to complications related to device implantation (Infection, hematoma, or other complication related to device implantation requiring intervention)

10.2.4 Supplementary Endpoints:

- Time to first acute (non-elective) hospitalization for stroke, systemic arterial embolism, transient ischemic attack, bleeding, cardiac disease, or complications, requiring an overnight stay in hospital or resulting in death
- Time to first admission for acute coronary syndrome or myocardial infarction
- Time to first implantation of cardiac pacemaker or defibrillator (with or without re-synchronization therapy)
- Time to first ablation of cardiac arrhythmia
- Development of AF burden
- Development of other cardiac arrhythmias
- ECG markers in predicting AF
- Changes in health economic costs
- Change from baseline QoL, and Quality Adjusted Life Years

- MRI: Change in occurrence of brain infarcts and white matter hyper-intensity
- MRI: Left atrial size, fibrosis, and function and the association with stroke and AF
- Safety aspects (such as infections) of ILR implants
- Inflammation markers and cardiac hormones and their prediction of AF and stroke
- Changes in cognitive dysfunction evaluated by MoCA forms
- Genetic prediction of AF and stroke
- Echocardiographic prediction of AF and stroke

10.2.5 Subsets:

Efficacy Analysis Set:

Efficacy analysis will be performed on the intent to treat (ITT) subject set, defined as comprising all subjects randomized. Efficacy analysis will include all available follow-up data from all randomized subjects. In addition sensitivity analysis may be carried out based on the un-treatment subject set, which is defined as subjects not receiving ILR regardless of randomization.

10.3 Sample Size Considerations:

We expect that 30% of the participants will have documented AF episodes lasting ≥6 minutes (and per protocol qualifying for OAC) during ILR monitoring for at least three years. The study population is expected to have a stroke rate of 0.7%/year in non-AF patients, and 2%/year in AF patients not treated with OAC. While we expect the number of AF-related strokes to be reduced by OAC, we assume that strokes unrelated to AF will not be influenced by this. We also include the assumption that 3% of all participants in the control group will become diagnosed with AF and start OAC. The annual rate of the primary end point in the control group is thus expected to be 1%. Finally, we expect that 5% in the ILR group will not be monitored due to refusal of ILR implantation or ILR explantation due to discomfort. These patients will participate at the same risk as patients in the control group and will be included in the analysis of the primary endpoint according to the intention-to-treat principle. We thus expect the overall hazard ratio to be 0.65. To analyse this reduction in the primary endpoint with a 2-sided α-level of 0.05 and a power of 80% in a 1:3 intervention:control-randomized population, we will need 279 primary events. Follow-up duration will be adjusted to accommodate the number of primary events. With an assumed length of inclusion period of 1-2 years and a median follow-up up 4 years, 6000 subjects will be needed.

10.4 Interim Analysis:

The likely hood of observing a statistically significant beneficial treatment effect is dependent on the event rate in the trial. When approximately 1/3 of the subjects have completed one year of follow-up this event rate will be discussed by the SC in order to decide, if the study should continue as planned.

The SC has the right to increase sample size or prolong follow-up if needed.

10.5 Access to Individual Subject Treatment Assignments:

Individual subject treatment assignments will be given to the SC if requested. Un-blinded (presented as group A and B) results, including aggregate and subject level data, can be presented to the SC for safety data reviews.

10.6 Planned Methods of Analysis:

10.6.1 General Approach/Considerations:

Baseline characteristics, demographics, pre-existing conditions, previous illnesses, and possible prior treatments will be summarized for each treatment group. Subjects disposition and reason for discontinuation will be summarized for each group. The primary and secondary efficacy endpoints are timed to event variables and survival techniques will be utilized to perform statistical estimation and hypothesis testing. Concomitant medications will be summarized for each treatment group and compared between groups to access their potential impact on the primary endpoint.

10.6.2 Analysis of Key Study Endpoints:

10.6.2.1 Primary Endpoint:

The principle analysis for the primary endpoints will employ the intend-to-treat principle, and use a survival analysis. To account for the competing risk of death, the cumulative incidences of the primary endpoint will estimated, plotted and group-wise compared in a multi-state fashion. A co-variate adjusted analysis of the combined primary endpoint using a cause-specific Cox proportional regression model will be performed as a supportive analysis. The hazard ratios and the corresponding 95% confidence intervals will be estimated. Subjects completing the study and not reaching the composite endpoint will be censored.

10.6.2.2 Secondary Endpoints:

See section 10.2.2.

For the time-to-event variables, analysis similar to the one employed for the primary endpoint will be used. The censoring mechanism will also be similar to the one used for the analysis of the primary endpoint. The secondary endpoints will be tested at a significance level of 0.05. Within the first two secondary endpoints the type 1 error rate associated with a multiplicity of comparisons will be controlled according to the Hochberg adjustment. The Quality of Life endpoint will be tested at the 5% significance level. Quality of Life score will be analysed as repeated measures analysis of covariance mixed effects model using baseline score as a covariate.

10.6.2.3 Safety Endpoint:

AEs: Subjects incidence rates of all AEs will be tabulated by system class,
preferred term, severity, and relationship to investigational product. Tables and/or narratives of "on-study" death, and serious and significant AEs, including those causing early withdrawal, will also be provided.

Complications from ILR implantation will be asked for and registered at all visits.

10.7 Study Oversight:

10.7.1 Steering Committee:

A SC comprising experts in the cardiovascular field will provide oversight and advice to ensure the most safe and efficient conduct and execution of the trial. The SC will consist of external physicians with experience and expertise in the design and conduct of cardiovascular clinical trials and will have representations from the working group on pacing and electrophysiology of the Danish Society of Cardiology, as well as representatives from Danish Stroke Neurologists. The main focus of the SC will be on the medical, ethical and scientific integrity of the study and on its timely completion. The SC will consider sample size re-examination in the eventuality at the observed, blinded pooled event rate for the primary endpoint falls below the assumptions from the design stage. SC members may be investigators in the study. To facilitate the communication of results from the study, a publication committee will be formed from the SC at an appropriate time, consisting of members of the SC and of others, who have contributed significantly to the study.

10.7.2 Endpoint Committee:

An endpoint committee (EC) will be formed prior to study commencement to adjudicate the primary endpoints in a blinded fashion (see Appendix B and F). A neurologist and a cardiologist are appointed in the EC.

With respect to correct classification of recorded AF events an “AF adjudication committee” will be established among the cardiologists in the SC. “The AF adjudication committee” will be composed of two cardiologist (rotation among SC members will occur). If these two cardiologists disagree a third cardiologist will be involved for final adjudication of AF.

11. INVESTIGATIONAL PRODUCT:

11.1 Loop recorders:

The ILR used in this study is the Reveal LINQ (Medtronic) or newer devices with similar functionality. The device is described in details in appendix E.

The device is capable of remote monitoring.

11.2 Compliance:

All patients randomized to ILR should undergo implantation. However, the number of patients randomized but not receiving an ILR will be registered as well as the number of explanted ILRs.

12. REGULATORY OBLIGATIONS:

12.1 Ethical considerations:

It is currently not known how asymptomatic AF should be treated. Only when all ("the majority of") AF is diagnosed the scientific and clinical problem about how to deal with asymptomatic AF can be addressed properly. One approach in this would be to perform continuous long-term monitoring of patients at risk. Therefore the LOOP study will answer clinically relevant questions and is based on this background.
In the present study patients will be randomized to either receive an ILR or not. For patients randomized to the control group, there is very little burden by the study since there is no placebo treatment. Patients randomized to ILR have the potential advantage of having AF detected and subsequently receiving appropriate anticoagulation therapy, which may turn out to be beneficial. However, such treatment may also cause potentially serious side effects.
Study patients randomized to the ILR group may have local complaints due to surgery and the insertion of a device subcutaneously. They will have a small scar after surgery. There is a small risk of infection in the region of surgery. They may have diagnosed atrial fibrillation (which is the purpose of the study) without symptoms and experience anxiety due to knowing that this was detected even though it does not give symptoms. They may start anticoagulation therapy and this may give side-effects such as bleedings, which would not have occurred if they had not participated in the study. Also, EGG artefacts may be misdiagnosed as atrial fibrillation.
Patients may experience that they become more focused on their health situation due to frequent data transmissions. On the other hand, patients may experience that it gives them a feeling of safety. Patients in the control group may feel unhappy since they are not followed as closely as they had hoped.

This study will require approval from the Regional Danish Committee on Biomedical Research Ethics, as well as allowance from The Danish Data Protection Agency. The Danish Medicine Agency has been asked if application for permission to perform the study was necessary, and the Agency has answered that an approval of the LOOP study was not necessary.

Overall the study addresses an important clinical problem and if the trial turns out positive it will improve therapy for a large number of patients. Since the socio-economic costs in therapy and care related to stroke are enormous the results from the study may also be important for society. Therefore, we consider the risks we impose on patients are acceptable.

12.2 Informed Consent:

An informed consent form is provided in Appendix H. Guidance for oral information of participants is given in Appendix I.

Before a subject's participation in the trial, the investigator is responsible for obtaining written informed consent from the subject after adequate explanation of the aims, methods, anticipated benefits, and potential hazards of the study, and the right to withdraw without consequences, before any protocol-specific screening procedures or any investigational products are administered. The potential benefit and hazard associated with participation in the study is described in the informed consent form (Appendix H). If the subject agrees, it is recommended that the investigator informs the subject's primary care physician of his/her participation in the clinical trial.

The acquisition of informed consent should be documented in the subjects medical records, and the informed consent form should be signed and personally dated by the subject, and by the person who conducted the informed consent discussion (not necessarily an investigator). The original signed informed consent form should be retained in accordance with institutional policy, and a copy of the signed consent form should be provided to the subject or the subject's legally acceptable representative.

If a potential subject is illiterate or visually impaired, the investigator must provide an impartial witness to read the informed consent form to the subject, and must allow for questions. Thereafter, both the subject and the witness must sign the informed consent form to attest that informed consent was freely given and understood.

12.3 Independent Ethics Committee:

A copy of the protocol, proposed informed consent form, other written subject information, and any proposed advertising material must be submitted to the Ethics Committee for the Capitol Region of Denmark. The investigator must submit and, when necessary, obtain approval from the Ethics Committee for all subsequent protocol amendments and changes to the informed consent document.

12.4 Subject Confidentiality:

The investigator must ensure that the confidentiality of a study subject is maintained. On the eCRFs or other documents mailed to the study office, subjects should be identified by their initials and a subject study number only. Documents that are not for submission (e.g. signed informed consent forms) should be kept in strict confidence by the investigator. In compliance with Federal regulations/ICH-GCP Guidelines, it is required that the investigator and institution permit authorized representatives of a monitoring company, and the EC, direct access to review a subjects original medical records for verification of study-related procedures and data. Direct access includes examining, analyzing, verifying, and reproducing any records and reports that are important to the evaluation of the study. The investigator is obligated to inform and obtain the consent of a subject for named representatives to have access to his/her study-related records without violating the confidentiality of the subject. All information obtained in relation to this protocol regarding participating patients is protected according to Danish law on Patients Legal Rights and Data Protection.

12.5 Investigator Signatory Obligations:

Each clinical study report should be signed by the investigator or the coordinating investigator. The coordinating investigator meets at least one of the following criteria:

- A recognized expert in the therapeutic area
- An investigator, who provided significant contributions to either the design or interpretation of the Study

13. ADMINISTRATIVE AND LEGAL OBLIGATIONS:

13.1 Protocol Amendments and Study Termination:

Protocol amendments must be made only with the prior approval of Steering Committee. Agreement from the investigator must be obtained for all protocol amendments and amendments to the informed consent document. The Ethics Committee must be informed of all amendments and give approval for any amendments likely to affect the safety of the subjects or the conduct of the trial. The Steering Committee reserves the right to terminate the study. The investigators reserve the right to withdraw from the study.

13.2 Study Documentation and Storage:

The investigator should maintain a list of appropriately qualified persons to whom he/she has delegated trial duties. All persons authorized to make entries and/or corrections on eCRFs will be included on the sponsor Delegation of Authority Form. Source documents are original documents, data, and records from which the subject's eCRF data are obtained. These include but are not limited to hospital records, clinical and office charts, laboratory and pharmacy records, diaries, microfiches, radiographs, and correspondence. CRF entries may be considered source data if the eCRF is the site of the original recording (i.e., there is no other written or electronic record of data). In this study, patient reported outcome information can be recorded directly on the web based CRF. It will be possible to perform electronic signature in the web based CRFs.The access to the web based CRFs will be protected by encryption.

The investigator and study staff are responsible for maintaining a comprehensive and centralized filing system of all study-related (essential) documentation, suitable for inspection at any time by representatives of the sponsor and/or applicable regulatory authorities. Elements should include:

- Subject files containing completed eCRFs, informed consents, and supporting copies of source documentation
- Study files containing the protocol with all amendments, investigator's brochure, copies of prestudy documentation (see section 12.3), and all correspondence to and from the EC.

In addition, all original source documents supporting entries in the eCRFs must be maintained and be readily available.

No study document should be destroyed without prior written agreement between the Steering Committee (or its appointed person) and the investigator.

13.3 Study Monitoring and Data Collection:

The Steering Committee representative and regulatory authority inspectors are responsible for contacting and visiting the investigator for the purpose of inspecting the facilities and, upon request, inspecting the various records of the trial (e.g., eCRFs and other pertinent data) provided that subject confidentiality is respected. The monitor appointed by the Steering Committee is responsible for inspecting the eCRFs at regular intervals as specified in the study monitoring plan, throughout the study to verify adherence to the protocol; completeness, accuracy, and consistency of the data; and adherence to local regulations on the conduct of clinical research. The monitor should have access to subject medical records and other study-related records needed to verify the entries on the eCRFs. The study is completed in accordance with this protocol and ICH-GCP guidelines. The investigator agrees to cooperate with the monitor to ensure that any problems detected in the course of these monitoring visits, including delays in completing eCRFs, are resolved. Inspection of site facilities and review of study-related records may occur to evaluate the trial conduct and compliance with the protocol, ICH-GCP, and applicable regulatory requirements.

All data should be entered into the web based CRF. The web based CRF should be completed when logged on to the system in the investigators own name.

Corrections in the electronic CRF are made electronically. The change must be initialised and dated by the investigator or a member of the study staff authorized by the investigator. Corrections to electronic forms will be automatically documented through the software's "audit trail".

To ensure the quality of clinical data across all subjects and sites, a clinical data management review will be performed on subject data received at the study office. During this review, subject data will be checked for consistency, omissions, and any apparent discrepancies. In addition, the data will be reviewed for adherence to the protocol and GCP. To resolve any questions arising from the clinical data management review process, data queries and/or site notifications will be sent to the site for completion and return to the study office.

The principal investigator will sign and date the indicated places on the eCRF. These signatures will indicate that the principal investigator inspected or reviewed the data on the case report form, the data queries, and the site notifications, and agrees with the content.

The study office will correct the database for the following CRF issues without notification to site staff:

- Misspellings, that do not change the meaning of the word (excluding AEs and medications)
- Location of data recorded on an incorrect CRF (e.g., moving lab data from general comments to the appropriate lab table)
- Obvious date errors
- Standard time to 24-hour clock errors

- Weight unit errors if a baseline weight has been established
- Height unit errors
- Administrative data (eg, event names for unscheduled visits or retests)
- Clarifying “other, specify” if data are provided (eg, race, physical exam)
- If both the end date and a status of continuing is indicated (eg, for AEs, concomitant medication, hospitalization) the end date will supersede
- Deletion of obvious duplicate data (eg, same results sent twice with the same date but different clinical planned events – week 4 and early termination)
- For AEs that record action taken code = 01 (none) and any other action code, 01 (none) may be deleted as it is superseded by other existing data
- If equivalent units or terms are recorded instead of the acceptable the sponsor standard (e.g., cc for mL, SQ for SC route, Not Examined for Not Done), the sponsor units or terms will be used
- If the answer to a YES or NO question is blank or obviously incorrect (e.g., Answers to the following questions do not reflect the data that are recorded or missing: Were there any AEs? Concomitant medications? Hospitalizations?)

All data will be kept for 15 years.

13.4 Language:

CRFs will be in English. Registered trade names for concomitant medications may be used instead of generic names. All written information and other material to be used by subjects and investigative staff must use vocabulary and language that are clearly understood.

13.5 Publication Policy:

To coordinate dissemination of data from this study, a publication committee consisting of the SC and selected participants is formed. The committee is expected to solicit input and assistance from other investigators as appropriate. Membership of the committee does not guarantee authorship—the criteria described below should be met for every publication. Authorship of any publications resulting from this study will be determined on the basis of the Uniform Requirement for Manuscripts Submitted to Biomedical Journals (International Committee of Medical Journal Editors, 1997) which states:
Each author should have participated sufficiently in the work to take public responsibility for the content. Authorship credit should be based on only substantial contributions to:

(a) Conception and design, or analysis and interpretation of data.
(b) Drafting the article or revising it critically for important intellectual content.
(c) Final approval of the version to be published.

Conditions (a), (b), and (c) must all be met.
All publications (e.g., manuscripts, abstracts, oral/slide presentations, book chapters) based on this study must be submitted to the Steering Committee for review.

The results from the study - both positive and negative as well as inconclusive results– will be submitted for publication in international peer-reviewed scientific journals.

13.6 Compensation:

Subjects will be covered by normal patient insurance.

14. REFERENCES:

1. Koch MB, Davidsen M, Juel K.
   Heart Diseases in Denmark: Incidence and future development. Report from the Danish Heart Foundation. October 2011.
   http://www.hjerteforeningen.dk/files/Rapporter_mm/Hjertekarsygdomme%20i%20Danmark.%20Forekomst%20og%20udvikling%202000-2009.pdf

2. Kirchhof P, Adamou A, Knight E, et al.
   How can we avoid a stroke crisis? ESC Working group report. Stroke prevention in atrial fibrillation. December 2009.
   http://www.escardio.org/communities/EHRA/publications/papers-interest/Documents/ehra-stroke-report-recommend-document.pdf

3. Camm AJ, Kirchhof P, Lip GY et al.
   Guidelines for the management of atrial fibrillation: The Task Force for the management of atrial fibrillation of the European Society of Cardiology.
   Europace. 2010; 12: 1360-1420.

4. Healey JS, Connolly SJ, Gold MR et al.
   Subclinical atrial fibrillation and the risk of stroke.
   NEJM 2012; 366: 120-9.

5. Savelieva I, Kakouros N, Kourliouros A, and Camm AJ.
   Upstream therapies for management of atrial fibrillation: review of clinical evidence and implications for European Society of Cardiology guidelines. Part I: primary prevention.
   Europace 2011; 13: 308-328.

6. De Jong AM, Maass AH, Oberdorf-Maass SU, Van Veldhuisen DJ, Van Gilst WH, Van Gelder IC.
   Mechanisms of atrial structural changes caused by stretch occurring before and during early atrial fibrillation.
   Cardiovasc Res 2010; 89: 754-765.

7. Nattel S, Burstein B and Dobrev D.
   Atrial Remodeling and Atrial Fibrillation: Mechanisms and Implications.
   Circ Arrhythmia Electrophysiol 2008; 1: 62-73.

8. Cheng S, Keyes MJ, Larson MG et al.
   Long-term outcomes in individuals with prolonged PR interval or first-degree atrioventricular block.
   JAMA 2009; 301: 2571-7.

9. Kolb C, Nürnberger S, Ndrepepa G et al.
   Modes of initiation of paroxysmal atrial fibrillation from analysis of spontaneously occurring episodes using a 12-lead Holter monitoring system.
   Am J Cardiol 2001; 88: 853-7.

10. Porsdal V, Boysen G.
Direct costs during the first year after intracerebral hemorrhage.
Eur J Neurol 1999; 6: 449-54.

11. Jørgensen HS, Nakayama H, Raaschou HO et al.
Acute stroke care and rehabilitation: an analysis of the direct cost and its clinical and social determinants. The Copenhagen Stroke Study.
Stroke 1997; 28: 1138-41.

12. Lunde L.
Can EQ-5D and 15D be used interchangeably in economic evaluations? Assessing quality of life in post-stroke patients.
Eur J Health Econ 2012 Jun 8. Epub ahead of print.

13. Larsen T, Olsen TS, Sorensen J.
Early home-supported discharge of stroke patients: A health technology assessment.
Int J Technol Assess Health Care 2006; 22: 313-20.

14. Pink J, Lane S, Pirmohamed M, Hughes DA.
Dabigatran etexialte versus warfarin in management of non-valvular atrial fibrillation in UK context: quantitative benefit-harm and economic analyses.
BMJ 2011 Oct 31;343:d6333.

15. Sorensen SV, Kansal AR, Connolly S et al.
Cost-effectiveness of dabigatran etexilate for the prevention of stroke and systemic embolism in atrial fibrillation: a Canadian payer perspective.
Thromb Haemost 2011; 105: 908-19.

16. You JH, Tsui KK, Wong RS, Cheng G.
Cost-Effectiveness of Dabigatran versus Genotype-Guided Management of Warfarin Therapy for Stroke Prevention in Patients with Atrial Fibrillation.
PLoS One 2012;7:e39640.

17. The CONSORT Group. http://www.consort-statement.org

18. Truelsen T, Piechowski-Jóźwiak B, Bonita R, Mathers C, Bogousslavsky J, Boysen G.
Stroke incidence and prevalence in Europe: a review of available data.
Eur J Neurol. 2006; 13: 581-98.

19. Knopman DS, Pennman AD, Catellier DJ, Coker LH et al.
Vascular risk factors and longitudinal changes on brain MRI - The ARIC study.
Neurology 2011; 76: 1879–1885,

20. Rostrup E, Gouw AA, Vrenken H, van Straaten ECW, Ropele S, Pantoni L, Inzitari D, Barkhof F, Waldemar G on behalf of the LADIS study group.
The spatial distribution of age-related white matter changes as a function of vascular risk factors—Results from the LADIS study.
NeuroImage 2012; 60: 1597-1607.

21. Pedersen KM.
DRG again again.
Ugeskr Laeger 2010; 172:2205.

22. Wittrup-Jensen KU, Lauridsen J, Gudex C et al.

Generation of a Danish TTO value set for EQ-5D health states.
Scand J Public Health 2009; 37: 459-66.

Supplementary references on the scientific themes:

Ahlsson A, Fengsrud E, Bodin L, Englund A.
Postoperative atrial fibrillation in patients undergoing aortocoronary bypass surgery carries an eightfold risk of future atrial fibrillation and a doubled cardiovascular mortality.
Eur J Cardiothorac Surg (2010), doi:10.1016/j.ejcts.2009.12.033

Botto GL, Padeletti L, Santini M, et al.
Presence and Duration of Atrial Fibrillation Detected by Continuous Monitoring: Crucial Implications for the Risk of Thromboembolic Events.
J Cardiovasc Electrophysiol, Vol. pp. 1-8.

Connolly SJ, Ezekowitz MD, Yusuf S et al.
Dabigatran versus Warfarin in Patients with Atrial Fibrillation.
N Engl J Med 2009;361:1139-51.

Hanke T, Charitos EI, Stierle U, et al.
Twenty-Four–Hour Holter Monitor Follow-Up Does Not Provide Accurate Heart Rhythm Status After Surgical Atrial Fibrillation Ablation Therapy. Up to 12 Months Experience With a Novel Permanently Implantable Heart Rhythm Monitor Device.
Circulation 2009;120[suppl 1]:S177–S184.

Huikuri H, Raatikainen MJP, Moerch-Joergens R, et al.
Prediction of fatal or near-fatal cardiac arrhythmia events in patients with depressed left ventricular function after an acute myocardial infarction.
European Heart Journal (2009) 30, 689–698. doi:10.1093/eurheartj/ehn537

Pedersen OD, Bagger H, Keller N, et al.
Efficacy of Dofetilide in the Treatment of Atrial Fibrillation-Flutter in Patients With Reduced Left Ventricular Function A Danish Investigations of Arrhythmia and Mortality ON Dofetilide (DIAMOND) Substudy.
Circulation 2001;104;292-296.

Sinha AM, Diener HC, Morillo CA, et al.
Cryptogenic Stroke and underlying Atrial Fibrillation (CRYSTAL AF): Design and rationale.
Am Heart J 2010;160:36-41.e1.

Pokushalov E, Romanov A, Corbucci G, et al.
Use of An Implantable Monitor to Detect Arrhythmia Recurrences and Select Patients for Early Repeat Catheter Ablation for Atrial Fibrillation: A Pilot Study.
Circ Arrhythm Electrophysiol. 2011 Dec;4(6):823-31. doi: 10.1161/CIRCEP.111.964809. Epub 2011 Sep 19.

Lamas G.
How much atrial fibrillation is too much atrial fibrillation?
N Engl J Med 2012 Jan 12;366(2):178-80. doi: 10.1056/NEJMe1111948.

Bloch Thomsen PE, Jons C, Raatikainen MJ, Moerch Joergensen R, et al.
Long-term recording of cardiac arrhythmias with an implantable cardiac monitor in patients with reduced ejection fraction after acute myocardial infarction: the Cardiac Arrhythmias and Risk Stratification After Acute Myocardial Infarction (CARISMA) study.

Circulation. 2010 Sep 28;122(13):1258-64. doi: 10.1161/CIRCULATIONAHA.109.902148. Epub 2010 Sep 13.

Buxton AE.
Implantable loop recorder in survivors of acute myocardial infarction: a glimpse of reality?
Circulation. 2010 Sep 28;122(13):1255-7. doi: 10.1161/CIRCULATIONAHA.110.976365. Epub 2010 Sep 13.

Heeringa J, van der Kuip DA, Hofman A, et al.
Prevalence, incidence and lifetime risk of atrial fibrillation: the Rotterdam study.
Eur Heart J. 2006 Apr;27(8):949-53. Epub 2006 Mar 9.

Brignole M, Vardas P, Hoffman E, et al.
Indications for the use of diagnostic implantable and external ECG loop recorders.
Europace. 2009 May;11(5):671-87. doi: 10.1093/europace/eup097.

Pedersen OD, Bagger H, Køber L, Torp-Pedersen C; TRACE Study Group.
Impact of congestive heart failure and left ventricular systolic function on the prognostic significance of atrial fibrillation and atrial flutter following acute myocardial infarction.
Int J Cardiol. 2005 Apr 8;100(1):65-71.

van Dam PM, van Oosterom A.
Analysing the potential of Reveal for monitoring cardiac potentials.
Europace. 2007 Nov;9 Suppl 6:vi119-23.

Pokushalov E, Taborsky M, Hindricks G., et al.
Variables Influencing Sensing after Reveal ImplanTation (VISIT).
Abstract presented at EURPACE 2009, 21 June - 24 June 2009, Berlin, Germany.

Friberg L, Hammar N, Pettersson H, Rosenqvist M.
Increased mortality in paroxysmal atrial fibrillation: report from the Stockholm Cohort-Study of Atrial Fibrillation (SCAF).
Eur Heart J. 2007 Oct;28(19):2346-53. Epub 2007 Aug 1.

Heeringa J, Conway DS, van der Kuip DA.
A longitudinal population-based study of prothrombotic factors in elderly subjects with atrial fibrillation: the Rotterdam Study 1990-1999.
J Thromb Haemost. 2006 Sep;4(9):1944-9. Epub 2006 Jul 5.

Petkar S, Hamid T, Iddon P.
Prolonged implantable electrocardiographic monitoring indicates a high rate of misdiagnosis of epilepsy--REVISE study.
Europace. 2012 Nov;14(11):1653-60. doi: 10.1093/europace/eus185. Epub 2012 Jun 28.

Zimetbaum P, Goldman A.
Ambulatory arrhythmia monitoring: choosing the right device.
Circulation. 2010 Oct 19;122(16):1629-36. doi: 10.1161/CIRCULATIONAHA.109.925610

Binici Z, Intzilakis T, Nielsen OW.
Excessive supraventricular ectopic activity and increased risk of atrial fibrillation and stroke.
Circulation. 2010 May 4;121(17):1904-11. doi: 10.1161/CIRCULATIONAHA.109.874982. Epub 2010 Apr 19.

Ravensbergen et al Regarding Article, “Long-Term Recording of Cardiac Arrhythmias
With an Implantable Cardiac Monitor in Patients With Reduced Ejection Fraction After Acute
Myocardial Infarction: The Cardiac Arrhythmias and Risk Stratification After Acute Myocardial

Infarction (CARISMA) Study" (Letter).
Circulation. 2012 Jan 3;125(1):e239

Friberg L, Hammar N, Rosenqvist M.
Stroke in paroxysmal atrial fibrillation: report from the Stockholm Cohort of Atrial Fibrillation.
Eur Heart J. 2010 Apr;31(8):967-75. doi: 10.1093/eurheartj/ehn599. Epub 2009 Jan 27.

Henriksson KM, Farahmand B, Asberg S, et al.
First-ever atrial fibrillation documented after hemorrhagic or ischemic stroke: the role of the CHADS(2) score at the time of stroke.
Clin Cardiol. 2011 May;34(5):309-16. doi: 10.1002/clc.20869. Epub 2011 Mar 13.

Kolominsky-Rabas PL, Heuschmann PU, Marschall D, et al.
Lifetime cost of ischemic stroke in Germany: results and national projections from a population-based stroke registry: the Erlangen Stroke Project.
Stroke 2006 May;37(5):1179-83. Epub 2006 Mar 30.

Edvardsson N, Frykman V, van Mechelen R, et al.
Use of an implantable loop recorder to increase the diagnostic yield in unexplained syncope: results from the PICTURE registry.
Europace. 2011 Feb;13(2):262-9. doi: 10.1093/europace/euq418. Epub 2010 Nov 19

Winkel TA, Rouwet EV, van Kuijk JP, et al.
Aortic surgery complications evaluated by an implanted continuous electrocardiography device: a case report.
Eur J Vasc Endovasc Surg. 2011 Mar;41(3):334-6. doi: 10.1016/j.ejvs.2010.11.006. Epub 2010 Dec 31.

Pokushalov E, Romanov A, Corbucci G, et al.
Ablation of paroxysmal and persistent atrial fibrillation: 1-year follow-up through continuous subcutaneous monitoring.
J Cardiovasc Electrophysiol. 2011 Apr;22(4):369-75. doi: 10.1111/j.1540-8167.2010.01923.x. Epub 2010 Oct 11.

Jons C, Jacobsen UG, Joergensen RM, et al.
The incidence and prognostic significance of new-onset atrial fibrillation in patients with acute myocardial infarction and left ventricular systolic dysfunction: a CARISMA substudy.
Heart Rhythm. 2011 Mar;8(3):342-8. doi: 10.1016/j.hrthm.2010.09.090. Epub 2010 Nov 18

Goetze JP, Friis-Hansen L, Rehfeld JF.
Atrial secretion of B-type natriuretic peptide.
Eur Heart J. 2006 Jul;27(14):1648-50. Epub 2006 Jun 19.

Hindricks G, Pokushalov E, Urban L, et al.
Performance of a new leadless implantable cardiac monitor in detecting and quantifying atrial fibrillation: Results of the XPECT trial.
Circ Arrhythm Electrophysiol. 2010 Apr;3(2):141-7. doi: 10.1161/CIRCEP.109.877852. Epub 2010 Feb 16.

El-Chami MF, Kilgo P, Thourani V, et al.
New-onset atrial fibrillation predicts long-term mortality after coronary artery bypass graft.
J Am Coll Cardiol. 2010 Mar 30;55(13):1370-6. doi: 10.1016/j.jacc.2009.10.058.

Pokushalov E, Romanov A, Cherniavsky A, et al.
Ablation of paroxysmal atrial fibrillation during coronary artery bypass grafting: 12 months' follow-up through implantable loop recorder.

Eur J Cardiothorac Surg. 2011 Aug;40(2):405-11. doi: 10.1016/j.ejcts.2010.11.083. Epub 2011 May 23.

Kirchhof P, Lip GY, Van Gelder IC, et al.
Comprehensive risk reduction in patients with atrial fibrillation: emerging diagnostic and therapeutic options—a report from the 3rd Atrial Fibrillation Competence NETwork/European Heart Rhythm Association consensus conference.
Europace. 2012;14(1):8-27

Fang MC, Go AS, Chang Y, et al.
Comparison of risk stratification schemes to predict thromboembolism in people with non-valvular atrial fibrillation.
J Am Coll Cardiol 2008;51(8):810-815

Camm AJ, Corbucci G, Padeletti L.
Usefulness of Continuous Electrocardiographic Monitoring for Atrial Fibrillation.
Am J Cardiol 2012; 110(2):270-276

Glotzer TV, Hellkamp AS, Zimmerman J, et al.
Atrial high rate episodes detected by pacemaker diagnostics predicts death and stroke.
Circulation 2003;107(12):1614-1619

Capucci A, Santini M, Padeletti L, et al.
Monitored atrial fibrillation duration predicts arterial embolic events in patients suffering from bradycardia and atrial fibrillation implanted with antitachycardia pacemakers.
J Am Coll Cardiol 2005; 46(10):1913–1920

Glotzer TV, Daoud EG, Wyse DG, et al.
The Relationship between Daily Atrial Tachyarrhythmia Burden From Implantable Device Diagnostics and Stroke Risk. The TRENDS Study.
Circ Arrhythmia Electrophysiol 2009;2(5):474-480

Boriani G, Botto GL, Padeletti L, et al.
Improving stroke risk stratification using the CHADS2 and CHA2DS2VASc risk scores in paroxysmal atrial fibrillation patients by continuous arrhythmia burden monitoring.
Stroke 2011;42(6):1768-1770

Santini M, Gasparini M, Landolina M, et al.
Device-detected atrial tachyarrhythmias predict adverse outcome in real-world patients with implantable biventricular defibrillators.
J Am Coll Cardiol 2011. 57(2):167-172

Welles CC, Whooley MA, Na B, Ganz P, Schiller NB, Turakhia MP.
The CHADS2 score predicts ischemic stroke in the absence of atrial fibrillation among subjects with coronary heart disease: Data from the Heart and Soul Study,
Am Heart J. 2011;162(3):555-561

Purerfellner H, Gillis AM, Holbrook R, Hettrick DA.
Accuracy of atrial tachyarrhythmia detection in implantable devices with arrhythmia therapies. Pacing Clin Electrophysiol. 2004; 27(7): 983–992

Lamas G.
How Much Atrial Fibrillation Is Too Much Atrial Fibrillation?
N Engl J Med 2012; 366(2):178-180

Boriani G., Santini M., Lunati M, .et al.
Improving Thromboprophylaxis Using Atrial Fibrillation Diagnostic Capabilities in Implantable Cardioverter-Defibrillators: The Multicentre Italian ANGELS of AF Project,
Circ Cardiovasc Qual Outcomes 2012; 5(2):182-188

van Elderen SG, de Roos A, de Craen AJ, et al.
Progression of brain atrophy and cognitive decline in diabetes mellitus: a 3-year follow-up.
Neurology. 2010 Sep 14;75(11):997-1002. doi: 10.1212/WNL.0b013e3181f25f06.

Knopman DS, Penman AD, Catellier DJ, et al.
Vascular risk factors and longitudinal changes on brain MRI: the ARIC study.
Neurology. 2011 May 31;76(22):1879-85. doi: 10.1212/WNL.0b013e31821d753f. Epub 2011 May 4.

Hajjar I, Quach L, Yang F, et al.
Hypertension, white matter hyperintensities, and concurrent impairments in mobility, cognition, and mood: the Cardiovascular Health Study.
Circulation. 2011 Mar 1;123(8):858-65. doi: 10.1161/CIRCULATIONAHA.110.978114. Epub 2011 Feb 14.

Portet F, Brickman AM, Stern Y, et al.
Metabolic syndrome and localization of white matter hyperintensities in the elderly population.
Alzheimers Dement. 2012 Oct;8(5 Suppl):S88-95.e1. doi: 10.1016/j.jalz.2011.11.007. Epub 2012 Jun 6.

Raz N, Yang Y, Dahle CL, et al.
Volume of white matter hyperintensities in healthy adults: contribution of age, vascular risk factors, and inflammation-related genetic variants.
Biochim Biophys Acta. 2012 Mar;1822(3):361-9. doi: 10.1016/j.bbadis.2011.08.007. Epub 2011 Aug 25.

Rostrup E, Gouw AA, Vrenken H, et al.
The spatial distribution of age-related white matter changes as a function of vascular risk factors--results from the LADIS study.
Neuroimage. 2012 Apr 15;60(3):1597-607. doi: 10.1016/j.neuroimage.2012.01.106. Epub 2012 Jan 28.

Seo SW, Lee JM, Im K, et al.
Cardiovascular risk factors cause cortical thinning in cognitively impaired patients: relationships among cardiovascular risk factors, white matter hyperintensities, and cortical atrophy.
Alzheimer Dis Assoc Disord. 2012 Apr-Jun;26(2):106-12. doi: 10.1097/WAD.0b013e31822e0831.

Gorelick PB, Scuteri A, Black SE, et al.
Vascular contributions to cognitive impairment and dementia: a statement for healthcare professionals from the american heart association/american stroke association.
Stroke. 2011 Sep;42(9):2672-713. doi: 10.1161/STR.0b013e3182299496. Epub 2011 Jul 21.

Zhu YC, Chabriat H, Godin O, et al.
Distribution of white matter hyperintensity in cerebral hemorrhage and healthy aging.
J Neurol. 2012 Mar;259(3):530-6. doi: 10.1007/s00415-011-6218-3. Epub 2011 Aug 30.

15. APPENDICES:

Appendix A – Study procedures – summary
Appendix B – Composition of Committees
Appendix C – Serious Adverse Events
Appendix D – Blood Samples for Genetic analysis and Blood Samples for Biomarkers
Appendix E – ILR information
Appendix F – Definition of composite endpoint
Appendix G – Funding
Appendix H – Informed consent
Appendix I – Guidelines for oral information

Appendix A – Study procedures – Summary
Study flow chart – a) ILR group

Phase	Screen	Rand.	Impl.	Follow-up						
**Visit**	**1**	**2**	**3**	**3A**	**4**	**4A**	**5**	**5A**	**6**	**6A**
**Month**	0,5	0	0,5		12,5	13-24	24,5	25-36	36,5	37-48
**Day**	-14	0	14		374		734		1094	
**Informed consent**	√									
**Medical history**	√				√		√		√	
**Concomitant Disease**	√				√		√		√	
**Concomitant Medication**	√				√		√		√	
**Demographics**	√									
**Echocardiography**		√					(√)		(√)	
**12-lead ECG**	√				√		√		√	
**Randomization**		√								
**Vital signs**	√				√		√		√	
**QoL and cognitive function**	√				√		√		√	
**Blood sample**	√									
**Adverse events**					√		√		√	
**MRI**		(√)		(√)		(√)				
**Health economics**					√		√		√	
**Remote ILR interrogation #**			√	√	√	√	√	√	√	√

# : only relevant for the ILR group; remote transmissions are sent automatically on a daily basis and routinely as patient activated every 4 weeks (to obtain ECG data during presumed sinus rhythm) during the entire study period

Appendix A – Study procedures – Summary
Study flow chart – b) Control group

Phase	Screen	Rand.	Impl.	Follow-up						
**Visit**	**1**	**2**	**3**	**3A**	**4**	**4A**	**5**	**5A**	**6**	**6A**
**Month**	0,5	0	0,5		12,5	13-24	24,5	25-36	36,5	37-48
**Day**	-14	0	14		374		734		1094	
**Informed consent**	√									
**Medical history**	√				√		√		√	
**Concomittant. Disease**	√				√		√		√	
**Concomittant Medication**	√				√		√		√	
**Demographics**	√									
**Echocardiography**		√					(√)		(√)	
**12-lead ECG**	√								√	
**Randomization**		√								
**Vital signs**	√								√	
**QoL and cognitive function**	√				√		√		√	
**Blood sample**	√									
**Adverse events**					√		√		√	
**MRI**		(√)		(√)		(√)				
**Health economics**					√		√		√	
**Remote ILR interrogation #**										

# : only relevant for the ILR group; remote transmissions are sent automatically on a daily basis and routinely as patient activated every 4 weeks (to obtain ECG data during presumed sinus rhythm) during the entire study period

Appendix B – Composition of Committees

Composition of the Steering committee (SC):

The SC, which consists of representatives from the founding partner institutions, will provide advice as to the project´s overall scientific quality and budget as well as concerning the balance between the project´s activities, goals and results.

The members of the Steering committee are:
Jesper Hastrup Svendsen, Rigshospitalet (chairman)
Lars Køber, Rigshospitalet
Søren Højberg, Bispebjerg Hospital
Ketil Haugan, Roskilde Hospital
Axel Brandes, Odense University Hospital
Christian Kronborg, University of Southern Denmark
Claus Graff, Aalborg University
Derk W. Krieger, Bispebjerg Hospital

Composition of the Event Committee (EC):

The event committee is responsible for evaluating and adjudicating events comprising all possible primary endpoints. The EC will classify deaths according to pre-specified criteria modified according to Hinkle and Thaler. Deaths will be classified as cardiovascular and non-cardiovascular. Cardiovascular will be divided into sudden and non-sudden cardiovascular deaths based on time specifications. Strokes will be divided into ischaemic and non-ischaemic strokes (bleeds) and non- classifiable strokes. The guidelines for classifications will be described in a separate charter.
There will be a neurologist and a cardiologist in the EC. The members of the EC are:

- Neurologist Professor Derk W. Krieger
- Cardiologist Professor Lars Køber

Composition of the International Advisory Committee (IAC):

An *International Scientific Advisory Group*, which will meet annually, will discuss issues of relevance for the project (theory, methods and results) and consist of internationally recognised experts in the field. The members of the IAC are:

- Professor Gregory Y.H. Lip, University of Liverpool, UK;
- Professor Mårten Rosenqvist, Karolinska Institute, Sweden; and
- Professor Dan Atar, University of Oslo, Norway.

They have all agreed to serve in the advisory group. All three are senior cardiologists with a strong scientific track record in atrial fibrillation.

Appendix C – Serious Adverse Events

Serious adverse events (SAE) are reported immediately in the electronic eCRFs. Every 6 month a summary report is generated to the Steering Committee. This report contains information on all SAEs. Since the study is performed in a study population with an increased risk of stroke and mortality SAE summaries will not be reported on a daily basis. An annual report will also be forwarded to the Ethics committee of the Capitol Region of Denmark. This report will be accompanied by a description of the events together with a statement regarding eventual consequences for the study.

Appendix D – Blood Samples for Genetic analysis and Blood Samples for Biomarkers

On inclusion, blood samples will be taken for routine measurement of electrolytes (Na and K), creatinine, haemoglobin concentration, white blood cells, platelets, troponins, pro-BNP and CRP (a total of 15 ml venous blood).

In addition, a total of 32 ml of venous blood is collected and stored in a research biobank.

The sampled blood for the research biobank is collected into a total of 6 vials:

A part of the blood is collected in 3 x 6 ml vials (purple coloured, K2EDTA) and stored as full-blood (a part is stored in vials containing RNAlater).

In addition, one vial containing 6 ml blood (purple coloured, K2EDTA) and 2 vials containing 4 ml blood (Z serum clot activator) are collected for centrifuging and after pipetting the supernatant is stored as plasma and serum, respectively.

Centrifuging is performed within 30 minutes after venous puncture and carried out at 3,000 RPM for 10 minutes. Samples are stored in freezer at minus 80 degrees Celcius.

Samples will be transferred to Rigshospitalet where they will be stored in the study related research biobank in a locked freezer. Surplus biological material from the research biobank is stored in a biobank for future research.

Appendix E – Device information: the Medtronic Reveal® LINQ:

In this study the Medtronic Reveal® LINQ insertable, cardiac monitor is used. The Reveal® LINQ is the newest and smallest ILR on the market which is expected to be market released by February 2014. It has battery longevity of three years and additionally 12 months of shelf life. Its size is 1.19 cc which is about 10% of the previous model (Medtronic Reveal® LINQ which has a volume of 9 cc). In contrast to the XT model the LINQ model has a build-in antenna which enables automatic remote transmissions. The device has an event memory of a total of 57 minutes (which is longer than the memory of the XT model: 49.5 minutes) (128 Hz) with up to 30 events in total. The total memory capacity is divided into 22.5 minutes of patient activated events (with the three most recent manual events lasting 7.5 minutes each), and additionally 27 minutes auto-activated events. The device is magnetic resonance (MR) conditional, which means that the Reveal® is safe for use in MR environments. Due to the algorithm the device uses for AF detection it will only categorize an arrhythmia event as atrial fibrillation, if it lasts more than two minutes in total. The arrhythmia detection capabilities of the Reveal® LINQ categorize arrhythmias into 5 groups: Fast VT, VT, bradycardia, asystolia, and AT/AF. The Reveal® LINQ Recorder is prepared for CareLink remote monitoring, with both automated daily transmissions and patient activated transmissions. The device can record AT/AF burden, heart rate variability, day versus night frequency and patient activity. Prior to implant it is recommended to check the vector of the device, with the purpose of finding the orientation with optimal R wave amplitude via the programmer. The device is supplied with a so-called patient assistant, which is a patient activating device. The device algorithm for atrial fibrillation detection is based on measurement of the RR-intervals and the delta RR-interval (change in RR interval from the preceding RR interval), which is afterwards visually presented in a so-called Lorenz plot (cluster signature matrix), where normal sinus rhythm or atrial flutter will be shown as a highly concentrated black spot in the center of the plot, whereas atrial fibrillation will be widely distributed in the cluster signature matrix (”gunshot profile”).

When patient activated episodes are stored, there is a total of 22.5 minutes for the episodes, and the device will always store the three last events, each lasting 7.5 minutes. Each of these 7.5 minute events will comprise 6.5 minutes prior to patient activation as well as 1 minute after patient activation. After patient activation a new episode will only be stored, if it happens more than 5 minutes after last activation. If the memory is full, the oldest episode will be overwritten. Per episode type there is a guaranteed storage of the three most resent episodes. When memory is full, the oldest stored ECGs will be removed. If data have been transmitted in the form of ECGs these will be stored forever as ECGs on Carelink. All episodes recorded by the ILR will be stored (as episodes with interpretation markers, but not necessarily as ECGs).

The system offers a visual indication to provide the patient with information that either everything is fine (no arrhythmia has been detected) (green lamp) or something with the heart rhythm needs action – please contact hospital (red lamp).

In the LOOP study it may seem reasonable to turn off one of the VT zones to increase the data storage.

In patients with frequent premature supraventricular contractions which the device incorrectly classify as atrial fibrillation, the device can be programmed to ignore these episodes by programming the function “ectopy rejection” “on” which will cause these episodes to be categorised as sinus rhythm. It is possible to bring the Carelink box to another country and make transmissions from abroad.

Suggested device programming of parameters:

a) FVT parameters are set at: 30/40 and a rate >222 bpm
b) VT parameters are set at: 24 consequtive beats at a rate > 182 bpm
c) Bradycardia is set at a rate < 30 bpm
d) Asystolia is set at a duration > 4.5 sec
e) Symptom activation is programmed on and programmed to 3 episodes of 7.5 min each
f) AT/AF is programmed at AF only on
g) Ectopy rejection is programmed off in standard settings (only programmed on in case it is observed that the device falsely categorises premature contractions as AF.

Since the LINQ Reveal has not yet been market released we do not fully know the ideal programming yet.

When the ILR reaches end of battery life the device is explanted. If a patient dies with an implanted ILR the device is explanted by a post-mortem procedure.

Appendix F – Definition of the composite endpoint

1: Stroke is defined as rapid onset of a focal/global neurological deficit, with duration ≥ 24 hours, or resulting in death, or supported by clear evidence of cerebral infarction on diffusion-weighted MRI imaging. There must be no other readily identifiable non-stroke cause for the clinical presentation.

Stroke is sub- categorized as follows:

-1a: Ischemic stroke, defined as stroke caused by lack of blood supply (e.g. thrombosis, embolism, systemic hypoperfusion or cerebral venous sinus thrombosis)

-1b: Hemorrhagic stroke, defined as a stroke caused by cerebral or subarachnoid hemorrhage.

-1c: Unspecified stroke, in case the patient did not undergo brain imaging or autopsy

2: Systemic arterial embolism include arterial embolic events that do not reach the brain; these may reach organs such as kidney, spleen, heart, intestine, eyes and extremities.

Adjudication is based on clinical evaluation of patient data.

Transient ischemic attack (TIA) is not a primary event, and is defined as a transient episode of neurologic dysfunction caused by focal brain, spinal cord, or retinal ischemia, **without** acute infarction.

Explanation: TIA was originally defined clinically by the temporary nature (<24 hours) of the associated neurologic symptoms. However, the arbitrary nature of the 24-hour time limit and lack of specific pathophysiologic meaning hampered the clinical and research utility of the term "TIA." Recognition of these problems led to a change to a tissue-based definition of TIA. The change was driven by advances in neuroimaging that enabled very early identification of ischemic brain injury and endorsed by 2009 guidelines from the American Heart Association and American Stroke Association (AHA/ASA).

Appendix G –Funding

Research grants have been obtained from the Danish National Foundation for Strategic Research (DKK 15.6 mio), the Research Foundation for the Capital Region of Denmark (DKK 2.0 mio) and the Danish Heart Foundation (DKK 150.000).
The first 600 implantable loop recorders(ILRs) used in the project will be purchased from the study funding from the Danish National Foundation for Strategic Research. The condition of this purchase has been altered so that the Reveal LINQ´s will be offered on the same terms as the original Reveal XT devices. The additional 900 Reveal LINQ ILRs will be donated from Medtronic as an unrestricted research grant (which has a value of DKK 5.4 mio (reduced price) or DKK 22.5 mio (ordinary list price)) and a grant to cover salary for research nurses at the hospitals (DKK 1.9 mio).
Medtronic has decided to support the LOOP study with an unrestricted research grant covering salaries for one research nurse for one year at each of the four participating hospitals (i.e. a total of four years of salary for research nurses; estimated salary per nurse including pension and vacation is 40,000 DKK per month which equals 480,000 DKK per year/nurse; i.e. a total of 1.920.000 DKK (the same salary estimate was used in the application to the Strategic Research Council).
Additional research grants will be applied for from private and public research foundations as well as from industry. Study participants and the Ethics committee will be informed about additional funding.
Patients will not receive compensation for their participation in the study, but may in selected cases receive compensation for their transport expenses.

Appendix H – Informed consent

SAMTYKKE ERKLÆRING OG FULDMAGT VDR. LOOP FORSØGET

- ✓ Jeg har modtaget og læst deltagerinformationen for dette forsøg, og min læge har besvaret alle mine spørgsmål vedrørende det videnskabelige forsøg.
- ✓ Jeg har haft tilstrækkelig tid til at overveje min deltagelse i forsøget, og jeg er opmærksom på at deltagelse i dette forsøg sker helt frivilligt.
- ✓ Jeg er klar over, at jeg kan afslutte min deltagelse på et hvilket som helst tidspunkt, uden at det påvirker kvaliteten af sundhedsvæsenets ydelser til mig.
- ✓ Jeg forstår, og er enig i, at mine personlige oplysninger bliver indsamlet fra mine medicinske journaler, brugt og behandlet (manuelt og computermæssigt) af de læger som er ansvarlige for undersøgelsen, eller alle andre udpegede, som indgår i dette forsøg (dvs. hospitaler, læger, lovgivende myndigheder, etiske komitéer).
- ✓ Jeg har modtaget en kopi af patientinformationen og denne samtykkeerklæring.

Vedrørende forskningsbiobank:
☐ Jeg giver tilladelse til at min blodprøve, må udtages og opbevares i en forskningsbiobank. Det biologiske materiale vil ikke uden fornyet godkendelse hos De Videnskabsetiske komiteer blive brugt i andre forskningsprojekter eller til andre formål og analyser, end dem, der er nævnt i informationen.

Du bedes tilkendegive om du ønsker din egen læge informeret om din deltagelse i forsøget:
☐ *Jeg ønsker min praktiserende læge skal informeres om min deltagelse i dette forsøg*
☐ *Jeg ønsker ikke min praktiserende læge skal informeres om min deltagelse i dette forsøg*

Hvis der kommer nye væsentlige helbredsoplysninger frem om dig i dette forsøg vil du blive informeret. Såfremt du ikke ønsker at modtage nye helbredsoplysninger bedes du markere her:
☐ (sæt x)

Ønsker du at blive informeret om forsøgets resultater samt eventuelle konsekvenser for dig?
☐ *Ja (sæt x)* ☐ *Nej (sæt x)*

Underskrift til samtykke til deltagelse i forsøget (hvis relevant):

______________________	______________________	______________________
Patientens navn	Underskrift	Dato

Erklæring fra forsøgslægen, forsøgssygeplejerske eller anden person udpeget af forsøgslederen, som afgiver informationen:
Jeg bekræfter, at jeg har forklaret indholdet af denne patientinformation og informeret samtykke for den ovennævnte person. Jeg har spurgt, om der er nogle spørgsmål, og jeg har besvaret alle spørgsmål, der er fremført.

______________________	______________________	______________________
Læge/sygeplejerske navn	Underskrift	Dato

Appendix I – Guidelines for oral information

Retningslinjer for mundtlig deltagerinformation.

Informationen gives af en person, som har de sundhedsfaglige forudsætninger for at kunne informere om forskningsprojektet og som har direkte tilknytning til dette, hvilket i praksis betyder at informationen gives af læge eller forskningssygeplejerske på et af de hospitaler der deltager i undersøgelsen.

Forsøgspersonerne er identificeret ved:

- Et registertræk hvor man har fundet personer der opfylder in- og eksklusionskriterier
- Personer der løbende identificeres på de deltagende hospitaler (i forbindelse med indlæggelse eller ambulant kontrol) og som opfylder in- og eksklusionskriterier
- Personer som henvender sig til hospitalerne fordi de ønsker at deltage og som opfylder in- og eksklusionskriterier (disse vil potentielt kunne komme fra andre end de 3 deltagende regioner)

De potentielle forsøgspersoner vil få følgende initiale skriftlige information:

LOOP forsøget

Titel: Sammenhængen mellem blodprop i hjernen og atrieflimren påvist ved langvarig registrering af hjerterytmen med loop recorder.

Med dette brev vil vi spørge, om du vil deltage i en forskningsundersøgelse. Forsøget har til formål at afklare, om man kan forebygge blodprop i hjernen ved at overvåge hjerterytmen med et lille apparat (en såkaldt loop recorder), der placeres under huden på forsiden af brystkassen. Nogle af de personer der deltager vil få placeret det lille apparat, mens andre ikke vil få dette (dette afgøres ved en lodtrækning). I forskningsundersøgelsen deltager i alt 6.000 patienter og af disse vil hver fjerde (dvs. 1.500) efter lodtrækning få placeret det lille apparat.

Vi har fundet frem til dig ved at søge i registre efter personer med adresse i Region Hovedstaden, Region Sjælland eller Region Syddanmark, der er fyldt 70 år og desuden har en eller flere af sygdommene: Forhøjet blodtryk, sukkersyge, hjertesvigt eller en tidligere blodprop i hjernen (”slagtilfælde”). Du kan også være blevet fundet i forbindelse med en nylig hospitalskontakt, eller ved at du har kontaktet os, fordi du har hørt om forsøget. Nogen gange kan der være fejl i registerdiagnoser, så hvis du fejlagtigt er blevet registreret med en af de nævnte diagnoser, beder vi dig orientere os herom.

Såfremt du allerede har fået påvist den hjerterytmeforstyrrelse, der kaldes atrieflimren kan du desværre ikke deltage. Du kan heller ikke deltage i forsøget, hvis du behandles med blodfortyndende medicin af typen Marevan, Pradaxa, Eliquis eller Xarelto

Hvis du er interesseret i at høre mere om forsøget kan du **kontakte projektsygeplejerske Sisse Lund (telefonnummer 35450686 eller e-mail: sisse.maj.lund.jensen@regionh.dk),** som vil sikre, at du kan deltage i forsøget.

Såfremt du ønsker at deltage, vil det være en god ide, hvis du kan have et familiemedlem eller en god ven med til samtalen på sygehuset.

Med venlig hilsen

Jesper Hastrup Svendsen
Professor, dr.med.

Appendix J – Endpoint Committee Charter

Clinical Endpoint Committee (CEC) Charter
The LOOP Study

Version 6.
Date: 20th October 2016
Revised on March 26, 2017

Members of the clinical endpoint committee (CEC):
Professor Lars Køber (chairman), Rigshospitalet, University of Copenhagen.
Professor Derk Krieger, University of Zürich.
The members of the CEC consist of experts in the field of cardiology or neurology with expertise in atrial fibrillation and stroke.
With respect to adjudication of atrial fibrillation episodes the CEC has given authority to members outside the committee to adjudicate atrial fibrillation episodes as described below.

1 **Purpose**
The committee will provide standardized adjudication of relevant protocol-defined events for the LOOP study. Events will include trial endpoints (excluding Quality of Life) and important specific adverse events. This charter describes the roles and responsibilities of the committee for adjudication of the primary endpoints. All members of the committee will adjudicate all endpoints that potentially could be a primary endpoint.

2 **Roles of CEC Members**
The primary roles of CEC members will be:
1. To review and provide input for this Charter;
2. To adjudicate all reported events as per this Charter;
3. To classify all endpoints into their specific type according to the set of definitions in this document.
Processes for adjudication and definitions for the events that will be adjudicated by the committee are described in this Charter. The committee will review and adjudicate all strokes and systemic embolisms and all deaths. The committee can include additional expertise as required for evaluation of events, and may obtain assistance in assessment of events.

3. **Reporting events to the Committee**
Potential endpoints will be reported in the eCRF, and supporting documentation (e.g., admission summaries, progress records, diagnostic test reports, consultant evaluations, discharge summaries, death certificates, autopsy reports) will be forwarded by each center to the Study Office at Rigshospitalet. The study administrator will gather all relevant material in patient specific binders, which will be used by the CEC. The study administrator will interact with study sites to collect additional documentation needed to prepare a complete document package for committee adjudication.

4. **Documentation for CEC adjudication**
Supporting documentation required for adjudication of an event may include but is not limited to the following:

- Emergency Department records
- Pertinent laboratory reports
- Selected reports from any other test performed to evaluate the patient
- Hospital admission notes and pertinent hospital record notes surrounding the event (physician notes and nursing notes)
- Selected consultant reports and pathology reports
- Hospital or Emergency Department discharge summaries
- Death/autopsy summary
- Selected relevant hospital records
- Narrative summary of the event

The supporting documentation will have all patient identifiers (name, CPR number and information about randomization) removed or completely obscured prior to submission to the study office and supplied with a study number.
Whenever possible, suspected endpoints will be presented to the CEC only when a complete package of information has been obtained (e.g., all appropriate eCRF information and an adequate set of supporting documentation).
Adjudicators will review the documentation as hard copies.

5. **Evaluation of the event by the Committee**
All cases of potential stroke, TIA or systemic embolism (or death) will be reviewed independently by two CEC members. If the committee members agree on the adjudication, the adjudication is considered complete. If the committee members disagree on adjudication, the event will be reviewed during a CEC meeting. If an event cannot be classified due to a lack of supportive documentation, the CEC will ask for additional information from the study coordinator. After all possible information is gathered, the CEC will render a final adjudication on the event. In certain death cases, only limited medical information may be available. CEC members should use their best clinical judgment when rendering a final decision based on available data and the event definitions within this Charter.

6. **Reporting of the Committee decision**
The completion of the adjudication eCRFs constitutes the committee decision. This decision will not be communicated to the sites before the end of the study.

7. **Definition of events for CEC**
Events requiring adjudication include:

- Stroke (or TIA)
- Systemic embolism
- Death

Source documentation will be reviewed for determination as to whether the event meets the classification criteria established in this charter. Decisions by the committee will be based on the documentation provided and good clinical judgment.
The CEC adjudication categories for study events are as follows. Further information for each is provided below.

ADJUDICATION OF STROKES

Brain imaging is required for all patients with symptoms of stroke or TIA to distinguish hemorrhagic from ischemic stroke.

1: Stroke is defined as the sudden onset of a focal neurologic deficit due to acute infarction of cerebrovascular cause lasting at least 24 hours or interrupted by death, and is categorized as follows:
-1a: Ischemic stroke, defined as stroke caused by lack of blood supply (e.g. thrombosis, embolism, systemic hypoperfusion or cerebral venous sinus thrombosis)
-1b: Hemorrhagic stroke, defined as a stroke caused by cerebral or subarachnoid hemorrhage.
-1c: Unspecified stroke, in case the patient did not undergo brain imaging or autopsy
Explanation: Above definitions have been used in the RE-LY, ARISTOTLE and ROCKET AF studies.

2: Transient ischemic attack (TIA) is defined as a transient episode of neurologic dysfunction caused by focal brain, spinal cord, or retinal ischemia, **without** acute infarction.

Explanation: TIA was originally defined clinically by the temporary nature (<24 hours) of the associated neurologic symptoms. However, the arbitrary nature of the 24-hour time limit and lack of specific pathophysiologic meaning hampered the clinical and research utility of the term "TIA." Recognition of these problems led to a change to a tissue-based definition of TIA. The change was driven by advances in neuroimaging that enabled very early identification of ischemic brain injury and endorsed by 2009 guidelines from the American Heart Association and American Stroke Association (AHA/ASA).

ADJUDICATION OF SYSTEMIC EMBOLISMS (SYSTEMIC EMBOLIC EVENTS; SEE)

SEE include arterial embolic events except those that reach the brain; SEE may reach organs such as kidney, spleen, heart, intestine, eyes and extremities.
Adjudication is based on clinical evaluation of patient data.

ADJUDICATION OF BLEEDING EPISODES

Major bleeding is defined according to the ISTH criteria as bleeding in a critical site (e.g. symptomatic intracranial bleeding (hemorrhagic stroke (see above) or subdural hemorrhage)), or clinically overt bleeding accompanied by a decrease in the hemoglobin level of at least 20 g per deciliter or transfusion of at least 2 units of packed red cells, or bleeding resulting in death. Nonmajor bleeding is defined as clinically overt bleeding that does not satisfy the criteria for major bleeding but leads to hospital admission, physician-guided medical or surgical treatment, or a change in antithrombotic therapy.

ADJUDICATION OF DEATHS

All deaths require adjudication by all committee members and classification as cardiovascular vs. non-cardiovascular and sudden vs. non-sudden. If the committee finds that a death is in fact caused by stroke, this will be registered as a primary endpoint in the eCRF.

Cardiovascular death includes

- Heart failure/cardiogenic shock (pump failure)
- Sudden cardiac death – witnessed
- Sudden cardiac death – unwitnessed
- Other cardiovascular causes (Acute coronary syndrome, cerebrovascular accident, Peripheral Vascular Disease, Systemic embolus, Pulmonary embolus)

- Cardiac or vascular procedure complication
- Other cardiovascular death, specify
- Presumed cardiovascular death (includes events classified as “unknown” cause of death)

Non-cardiovascular death includes
- Renal Failure
- Pulmonary (other than pneumonia)
- Pneumonia
- Sepsis
- Other infection
- Gastrointestinal (including GI hemorrhage)
- Seizure (not related to cerebrovascular accident, CVA)
- Other neurologic
- Hematologic
- Malignancy
- Non-cardiovascular procedure complication

Definitions used to classify cardiovascular deaths

Death due to Heart Failure/Cardiogenic Shock (Pump failure):
Death occurring in the context of clinically worsening symptoms and/or signs of heart failure without evidence of another cause of death. New or worsening signs and/or symptoms of congestive heart failure (CHF) may include any of the following:
- New or increasing symptoms and/or signs of heart failure (such as dyspnea, fatigue, edema) requiring the initiation of, or an increase in, treatment directed at heart failure or occurring in a patient already receiving maximal therapy for heart failure
- Heart failure symptoms or signs requiring continuous intravenous therapy or oxygen administration
- Confinement to bed predominantly due to heart failure symptoms
- Pulmonary edema sufficient to cause tachypnea and distress not occurring in the context of an acute myocardial infarction or as the consequence of a primary arrhythmic event
- Cardiogenic shock, manifest as clinical signs and symptoms of hypoperfusion assumed to be secondary to cardiac dysfunction, and not occurring in the context of an acute myocardial infarction or as the consequence of a primary arrhythmic event
- Patients who are hospitalized and are being actively treated for heart failure and who have a sudden death as the terminal event will be classified as having a heart failure related death.

Sudden Presumed Cardiac Death:
Death that occurs unexpectedly in a previously stable patient will be adjudicated as witnessed or unwitnessed sudden cardiac deaths:
Witnessed sudden cardiac death:
- Witnessed and instantaneous without new or worsening symptoms
- Witnessed within 60 minutes of the onset of new or worsening cardiac symptoms
- Witnessed and attributed to an identified arrhythmia (e.g., captured on an electrocardiographic (ECG) recording or witnessed on a monitor by either a medic or paramedic or found by interrogation of a device (ILR/ICD/CRT/pacemaker)

- Subjects unsuccessfully resuscitated from cardiac arrest or successfully resuscitated from cardiac arrest but who die without identification of a non-cardiac etiology
  Note that if a witnessed sudden cardiac death occurs as a complication of another primary cardiac process, eg, cardiogenic shock or acute myocardial infarction, the primary process will be adjudicated as the cause of death.

Unwitnessed Sudden Presumed Cardiac Death:
An unwitnessed death is one that occurs in a patient who, when last seen alive, within an observation period of 72 hours:
- Did not manifest another life-threatening non-cardiac disease (e.g., infectious, metabolic disorders);

and/or
- Did not reveal a cause other than cardiovascular (e.g., trauma) at the scene of death;

and/or
- Death was ruled cardiovascular in cause on an autopsy report or death certificate, and occurred in the absence of pre-existing circulatory failure or other modes of death.

If an unwitnessed sudden cardiac death occurs, information regarding the patient's clinical status within the week preceding death should be reviewed for potential insight into the primary cause of death.

Non-Sudden Death due to Other Cardiovascular Causes:
These are defined from medical records and include acute coronary syndrome, cerebrovascular accident (stroke), peripheral vascular disease, systemic embolus, pulmonary embolus, cardiac or vascular procedure complication, other specified cardiovascular death

Non-Sudden Presumed Cardiovascular Death:
All deaths not attributed to the categories of cardiovascular death and not attributed to a non-cardiovascular cause, are presumed cardiovascular deaths and as such are part of the cardiovascular mortality endpoint.

Definitions used to classify non-cardiovascular deaths
Non-cardiovascular death is defined as any death not covered by cardiac death or vascular death and includes the following:
- Renal Failure
- Pulmonary (other than pneumonia)
- Pneumonia
- Sepsis
- Other infection
- Gastrointestinal (including GI hemorrhage)
- Seizure (not related to CVA)
- Other neurologic
- Hematologic
- Malignancy
- Non-cardiovascular procedure complication
- Other (specify)

Non CV death will primarily be reported as yes or no. In cases where patients are admitted to hospital for a cardiovascular reason and dies with an infection the event will be classified as CV death.
