# Supplementary material for: Prevalence and Prognostic Significance of Bradyarrhythmias in Patients Screened for Atrial Fibrillation vs Usual Care: Post Hoc Analysis of the LOOP Randomized Clinical Trial
Source: JAMA Cardiol. 2023 Feb 15;8(4):326–34. doi: 10.1001/jamacardio.2022.5526 (PMC9932940; doi:10.1001/jamacardio.2022.5526)
Supplement: Supplement 2. — eFigure 1. Cumulative incidence of sudden cardiovascular death by randomization arm eTable 1. Concomitant diagnoses of bradyarrhythmia and atrial fibrillation eTable 2. Baseline variables associated with incident bradyarrhythmia [file jamacardiol-e225526-s002.pdf]

# Supplemental Online Content

Diederichsen SZ, Xing LY, Frodi DM, et al. Prevalence and prognostic significance of bradyarrhythmias in patients screened for atrial fibrillation vs usual care: post hoc analysis of the LOOP randomized clinical trial. *JAMA Cardiol*. Published online February 15, 2023. doi:10.1001/jamacardio.2022.5526

**eFigure.** Cumulative incidence of sudden cardiovascular death by randomization arm

**eTable 1.** Concomitant diagnoses of bradyarrhythmia and atrial fibrillation

**eTable 2.** Baseline variables associated with incident bradyarrhythmia

This supplemental material has been provided by the authors to give readers additional information about their work.

© 2023 Diederichsen SZ et al. *JAMA Cardiology.*

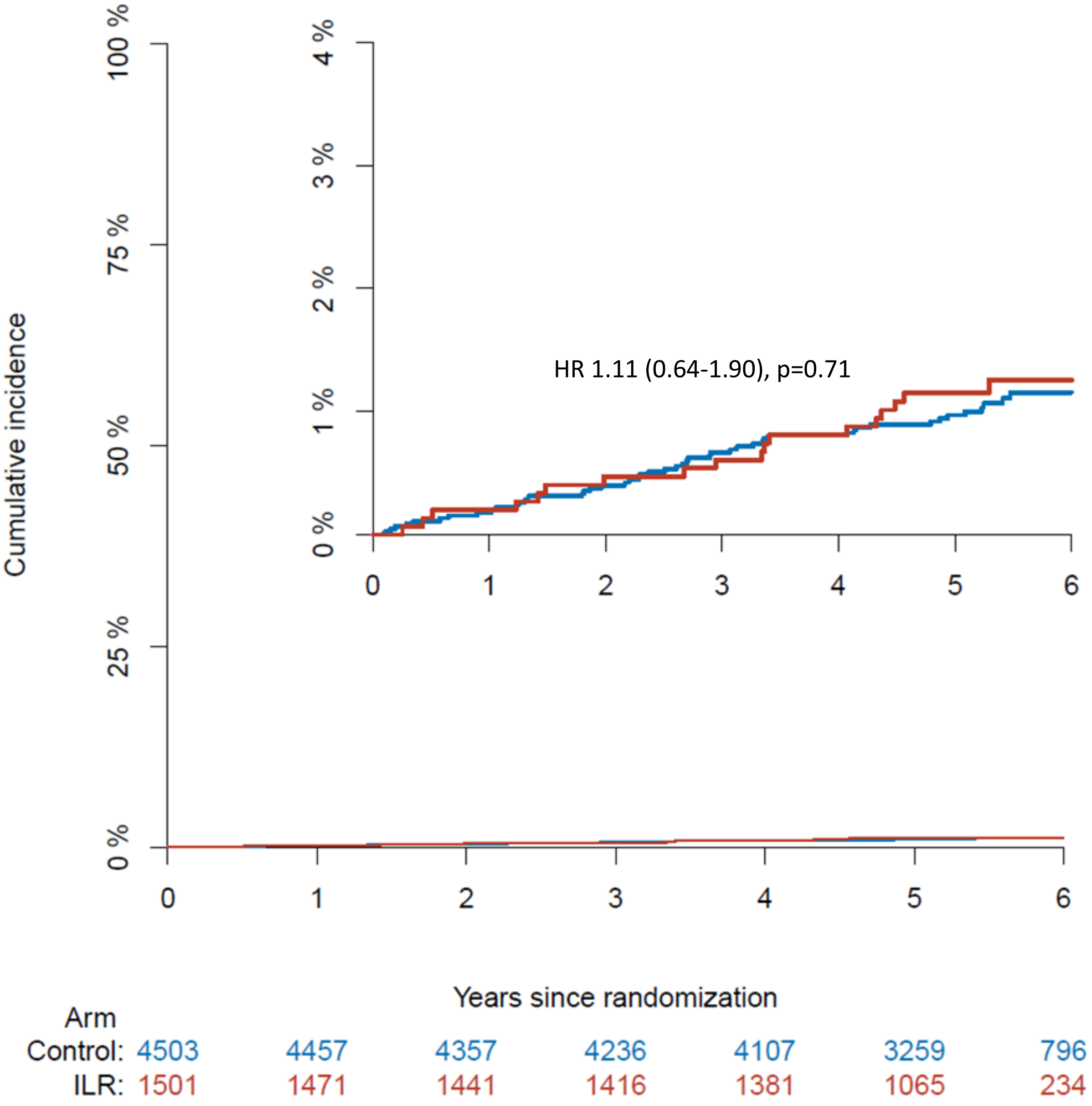


**eFigure. Cumulative incidence of sudden cardiovascular death by randomization arm**

The figure shows time-to-first-event curves for diagnosis of sudden cardiovascular death by randomization group. ILR, implantable loop recorder

© 2023 Diederichsen SZ et al. *JAMA Cardiology.*

**eTable 1. Concomitant diagnoses of bradyarrhythmia and atrial fibrillation**

| | Control, n=4503 | | | | ILR, n=1501 | | | |
|---|---|---|---|---|---|---|---|---|
| | AF | | No AF | All | AF | | No AF | All |
| Brady | 68 (12.4) | 30 (5.45)*<br>38 (6.91)** | 104 (2.63) | 172 (3.82) | 128 (26.8) | 54 (11.3)*<br>74 (15.5)** | 184 (18.0) | 312 (20.8) |
| No brady | 482 (87.6) | | 3849 (97.4) | 4331 (96.2) | 349 (73.2) | | 840 (82.0) | 1189 (79.2) |
| All | 550 (100) | | 3953 (100) | 4503 (100) | 477 (100) | | 1024 (100) | 1501 (100) |

Values are presented as number (percent). *Bradyarrhythmia before AF diagnosis; ** Bradyarrhythmia after AF diagnosis

The table shows the number and column-wise percentages of participants with and without bradyarrhythmia (sinus node dysfunction or atrioventricular block) according to AF diagnosis during follow-up. Chi-squared tests revealed significantly different distributions in both randomization groups. AF, atrial fibrillation; brady, bradyarrhythmia

© 2023 Diederichsen SZ et al. *JAMA Cardiology.*

**eTable 2.** **Baseline variables associated with incident bradyarrhythmia**

| | Control | | | ILR | | |
|---|---|---|---|---|---|---|
| | **HR** | **95% CI** | **p-value** | **HR** | **95% CI** | **p-value** |
| Age, per year | 1.08 | (1.04-1.11) | <0.0001 | 1.03 | (1.01-1.06) | 0.014 |
| Male sex | 2.14 | (1.51-3.03) | <0.0001 | 1.51 | (1.19-1.92) | <0.001 |
| Hypertension | 0.91 | (0.56-1.47) | 0.69 | 1.21 | (0.78-1.87) | 0.40 |
| Diabetes | 1.34 | (0.96-1.88) | 0.087 | 1.13 | (0.88-1.45) | 0.35 |
| Heart failure | 1.72 | (1.04-2.85) | 0.036 | 0.94 | (0.55-1.59) | 0.81 |
| Prior stroke, TIA, or SAE | 0.79 | (0.55-1.15) | 0.22 | 1.15 | (0.89-1.49) | 0.28 |
| Prior CABG | 1.73 | (1.10-2.73) | 0.018 | 1.45 | (0.95-2.20) | 0.086 |
| Valvular heart disease | 2.10 | (1.24-3.54) | 0.0055 | 1.10 | (0.65-1.85) | 0.73 |
| Prior syncope | 1.44 | (1.02-2.04) | 0.039 | 1.50 | (1.16-1.94) | 0.0022 |
| Body mass index, per kg/m$^2$ | 1.03 | (0.99-1.06) | 0.13 | 1.04 | (1.01-1.06) | 0.0029 |
| Resting sinus rate, per 10 bpm | 0.83 | (0.73-0.94) | 0.0046 | 0.92 | (0.84-1.01) | 0.093 |

The table shows the output of multivariate cause-specific Cox proportional hazard models of time from randomization to bradyarrhythmia diagnosis in each randomization group, respectively. bpm, beats per minute; CABG, coronary artery bypass graft; HR, hazard ratio; ILR, implantable loop recorder; SAE, systemic arterial embolism; TIA, transient ischemic attack

© 2023 Diederichsen SZ et al. *JAMA Cardiology.*
