# Supplementary material for: Prevalence and Prognostic Significance of Bradyarrhythmias in Patients Screened for Atrial Fibrillation vs Usual Care: Post Hoc Analysis of the LOOP Randomized Clinical Trial
Source: JAMA Cardiol. 2023 Feb 15;8(4):326–34. doi: 10.1001/jamacardio.2022.5526 (PMC9932940; doi:10.1001/jamacardio.2022.5526)
Supplement: Supplement 3. — Data sharing statement [file jamacardiol-e225526-s003.pdf]

# Data Sharing Statement

Diederichsen. Prevalence and Prognostic Significance of Bradyarrhythmias in Patients Screened for Atrial Fibrillation vs Usual Care. *JAMA Cardiol*. Published February 15, 2023. doi:10.1001/jamacardio.2022.5526

**Data**
**Data available:** Yes
**Data types:** Other (please specify)
**Additional Information:** The data will be available to participants in future individual-level metaanalyses (afscreen.org and AFFECT-EU)
**How to access data:** soeren.zoega.diederichsen@regionh.dk
**When available:** beginning date: 12-31-2022

**Supporting Documents**
**Document types:** Statistical/analytic code
**How to access documents:** Email to soeren.zoega.diederichsen@regionh.dk
**When available:** With publication

**Additional Information**
**Who can access the data:** Researchers whose proposed use of the data has been approved
**Types of analyses:** Statistical/analytic code can be shared with the above mentioned researchers for any purpose
**Mechanisms of data availability:** Email to soeren.zoega.diederichsen@regionh.dk
